# Efficient homing of T cells via afferent lymphatics requires mechanical arrest and integrin-supported chemokine guidance

**DOI:** 10.1038/s41467-020-14921-w

**Published:** 2020-02-28

**Authors:** Rieke Martens, Marc Permanyer, Kathrin Werth, Kai Yu, Asolina Braun, Olga Halle, Stephan Halle, Gwendolyn E. Patzer, Berislav Bošnjak, Friedemann Kiefer, Anika Janssen, Michaela Friedrichsen, Jenny Poetzsch, Karan Kohli, Yvonne Lueder, Rodrigo Gutierrez Jauregui, Nadine Eckert, Tim Worbs, Melanie Galla, Reinhold Förster

**Affiliations:** 1 Institute of Immunology, Hannover Medical School, Hannover, Germany; 2 Mammalian Cell Signaling Laboratory, Max Planck Institute for Molecular Biomedicine, Münster, Germany; 3 Institute of Experimental Hematology, Hannover Medical School, Hannover, Germany; 4 Cluster of Excellence RESIST (EXC 2155), Hannover Medical School, Hannover, Germany; 5 Present Address: Department of Biochemistry and Molecular Biology, Monash University, Clayton, Victoria Australia; 6 Present Address: Fred Hutchinson Cancer Research Center, Seattle, WA USA

**Keywords:** Chemokines, Imaging the immune system, Lymphatic system, Lymph node

## Abstract

Little is known regarding lymph node (LN)-homing of immune cells via afferent lymphatics. Here, we show, using a photo-convertible Dendra-2 reporter, that recently activated CD4 T cells enter downstream LNs via afferent lymphatics at high frequencies. Intra-lymphatic immune cell transfer and live imaging data further show that activated T cells come to an instantaneous arrest mediated passively by the mechanical 3D-sieve barrier of the LN subcapsular sinus (SCS). Arrested T cells subsequently migrate randomly on the sinus floor independent of both chemokines and integrins. However, chemokine receptors are imperative for guiding cells out of the SCS, and for their subsequent directional translocation towards the T cell zone. By contrast, integrins are dispensable for LN homing, yet still contribute by increasing the dwell time within the SCS and by potentially enhancing T cell sensing of chemokine gradients. Together, these findings provide fundamental insights into mechanisms that control homing of lymph-derived immune cells.

## Abstract (web summary)

Immune cells mostly enter lymph nodes (LN) from blood circulation, but whether afferent lymphatics contributes to LN entry is unclear. Here, the authors show, using a photo-convertible reporter, that T cells in afferent lymphatics frequently enter LN and become arrested in the subcapsular sinus, with chemokines and integrins further guiding their migration in the LN.

## Introduction

Lymph nodes (LNs) integrate all components required for immune responses as assembly points for immune cells as well as as filter systems for antigens^1,2^. Lymphocytes enter LNs from blood via specialized high endothelial venules (HEV) or via afferent lymphatic vessels. Afferent lymph vessels drain lymph fluid and cells from the periphery into the subcapsular sinus (SCS) of LNs. The SCS lumen is confined by a collagen IV-rich capsule, a layer of ceiling lymphatic endothelial cells (LECs) to the outside, and a layer of floor LECs towards the parenchyma. Reticular fibers that span from the capsule to the sinus floor and marginal reticular cells (MRCs) located in the abluminal side of the SCS potentially stabilize the SCS^3–7^. The SCS extends into the medullary sinus region from where efferent lymph vessels exit LNs^8^. LN homing of immune cells via HEV has been characterized in great detail^1,2,9^, whereas the molecular and cellular mechanisms underlying homing via afferent lymphatics are poorly understood. We have previously described the adoptive intra-lymphatic (i.l.) transfer of immune cells in mice as an approach to identify routes and molecules involved in the homing process of lymph-derived cells into LNs^10^. Intra-lymphatically transferred dendritic cells (DCs) and naive T cells use different entry routes into the LN parenchyma. While DCs transmigrate directly through the afferent side SCS floor, naive T cells enter the T cell-rich paracortex from the medullary sinus system in a retrograde manner^10^.

The chemokine receptor CCR7 has been identified as one of the key regulators of immune cell entry into LNs. LN homing of naïve T cells, central memory T cells, B cells, and plasmacytoid DCs via HEV as well as LN entry of afferent lymph-derived DCs depend to a large degree on signals mediated via CCR7 (refs. ^10–15^). Another chemokine receptor, CCR8, was described to facilitate LN metastasis of lymph-derived tumor cells^6^. In this model, activation of CCR8 on tumor cells is mediated by CCL1 expressed on SCS endothelial cells in LNs allowing tumor cell entry from the collecting lymphatic vessel into the SCS. Moreover, the type 2 transmembrane glycoprotein plasmalemma vesicle-associated protein (PLVAP) has previously been suggested to control entry of lymphocytes and antigens into LNs^16^. As described for tissue-derived DCs, activated T cells are also known to enter LNs via afferent lymph vessels^11,17,18^. In addition, effector T cells leaving the LN via efferent lymph pass several LNs before reaching the blood^8,17^ each time deciding to bypass or enter the LN parenchyma. Little is known about their fate when reaching the SCS and about the routes and mechanisms allowing activated, lymph-derived (ald) T cells to enter the paracortical T cell zone (TCZ) within the LN parenchyma.

Recirculation of lymphocytes through LNs is essential for immune homeostasis. However, the mechanisms regulating lymphocyte homing via afferent lymphatics are not completely defined. In the present study, using high-resolution imaging and i.l. transfer of in vitro- or in vivo-activated CD4 T cells, we find that arriving cells are initially retained in the SCS primarily by means of a mechanical barrier consisting of a three-dimensional (3D) sieve of cells and fibers within this sinus space. Crawling of ald T cells on the SCS neither relies on integrin nor chemokine receptor signaling. However, chemotactic signals are essentially required for their subsequent translocation into the LN parenchyma that occurs through preformed pores amply present in the basal membrane of the SCS floor. We also show that T cells migrate within the parenchyma in the absence of any integrins, although these molecules facilitate migration velocity and T cell translocation. Thus, our study describes the molecular and cellular events that control LN entry of lymphocytes that recirculate through afferent lymphatics.

## Results

### Activated CD4 T cells recirculate to downstream LNs in vivo

**Fig. 1:**
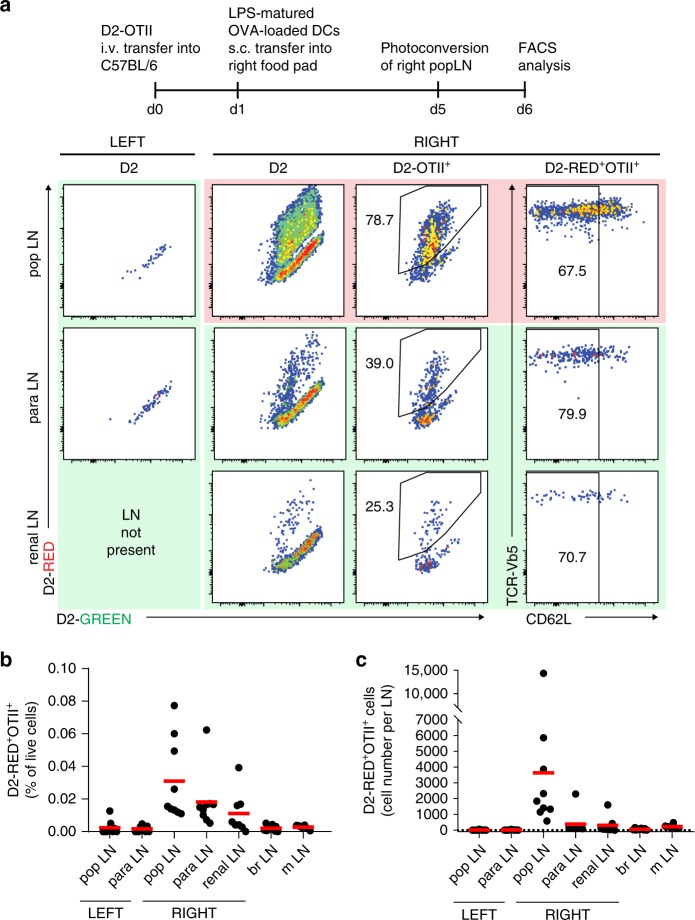
Recently activated CD4 cells home to downstream LNs in vivo. **a** Experimental setup to investigate lymphocyte migration through lymphatic vessels. Recipient B6 mice received OTII^+^ cells expressing the green-to-red photo-convertible fluorescent protein Dendra2 (D2) and were immunized with LPS-matured OVA-loaded DCs into the right foodpad. Five days later, the right pop LN was photo-converted by exposure to UV light (see also Supplementary Fig. 1). Representative FACS plots from the left non-photo-converted (green background) and right photo-converted (red background) pop LNs and from non-photo-converted (green background) downstream para-aortic and renal LNs analyzed 24 h after UV exposure. Plots are gated on D2, D2-OTII^+^, or D2-RED^+^OTII^+^ cells as indicated. **b** Frequency of D2-RED^+^OTII^+^ cells among all living cells and **c** absolute cell counts of D2-RED^+^OTII^+^ cells per LN (pop popliteal, para para-aortic, br brachial, m mesenteric, D2 Dendra2). Representative (**a**) or pooled (**b, c**) data of nine mice analyzed in three independent experiments.

It has been known for decades that activated T cells arrive via afferent lymphatic vessels in LNs^17,18^, but the mechanisms of LN entry and the molecules involved are poorly understood. We first created a mouse strain, in which a stop-flox-cassette, encoding a fusion protein of the photo-convertible protein Dendra2 (D2) and histone H2B^19,20^ was placed in the *Rosa-26* locus (for details see Supplementary Fig. 1). These mice were crossed to the F1 offspring of *Vav-iCre* and mice carrying a transgenic T cell receptor recognizing the ovalbumin (OVA) 323–339 MHC class II epitope (OTII mice). Lymphocytes from these OTII-Dendra2:OT2 mice were i.v. transferred into C57BL/6 recipients. The next day, these animals were immunized s.c. in the right footpad using LPS-matured OVA-loaded bone marrow-derived DCs. Four days later, the right popliteal (pop) LN was illuminated through the intact skin for 90 s with UV light that converts the green fluorescent protein Dendra2 (D2-GREEN) into a red fluorescent protein (D2-RED). This exposure time was chosen based on our preliminary in vitro observations showing that this amount was optimal to consistently photo-convert all D2-expressing cells into D2-RED cells (Supplementary Fig. 2). While OTII-D2 cells harvested from non-UV-exposed pop LNs immediately after illumination emitted D2-GREEN fluorescent light, OTII-D2 cells residing in UV-exposed popliteal LNs showed a strong D2-RED fluorescence (see Methods for details), demonstrating that all D2-expressing cells residing in a reactive LN can be photo-converted through the intact skin. Analysis of LNs 24 h after UV exposure revealed that approx. 75% of OTII-D2 cells located in the photo-converted pop LN remained D2-RED fluorescent (Fig. 1a). Notably, D2-RED^+^ OTII cells were also present at high frequency in LNs downstream of the UV-exposed right pop LN such as the right para-aortic or the right renal LNs but not in other LNs such as the contralateral, left pop. or para-aortic LN (Fig. 1a, b). As expected, the LNs positioned downstream of the photo-converted LN contained higher numbers of D2-RED^+^OTII cells than non-downstream LNs (Fig. 1c). We also noted that photo-converted LNs contained approximately 10-fold more D2-RED^+^OTII cells than LNs positioned downstream, while the percentage of D2-RED^+^OTII was similar at both locations. The reason for similar frequencies of D2-RED^+^OTII cells in UV-exposed pop LNs vs. none exposed downstream LNs remains unknown but most likely is incidental. Further analysis of these D2-RED cells revealed an activated phenotype since most of them lacked expression of L-selectin (Fig. 1a, right panel). These data demonstrate that CD4 T cells recently activated in the pop LN can home with high efficacy into downstream LNs.

### CD4 T cells enter the LN parenchyma through the SCS floor

**Fig. 2:**
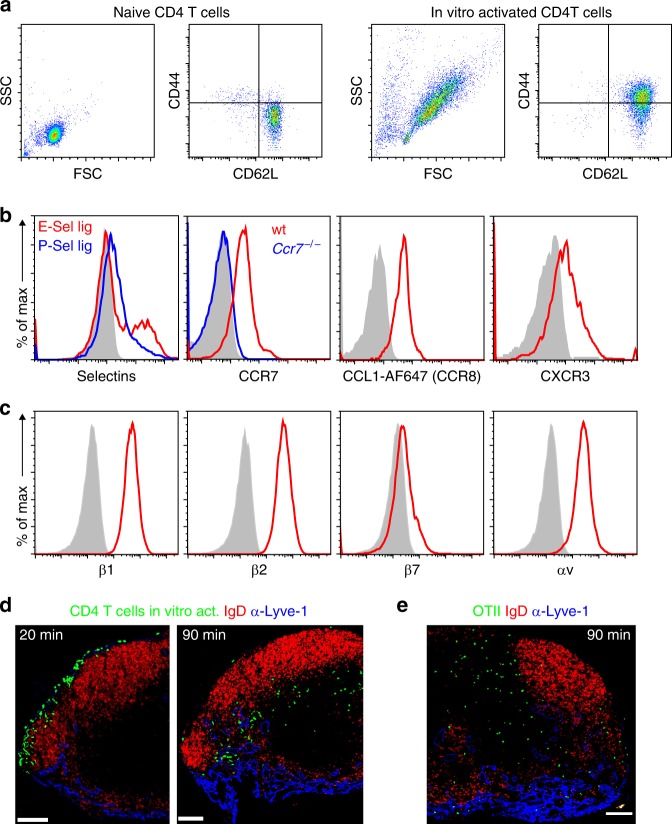
Lymph-derived activated CD4 T cells enter LN parenchyma via the subcapsular sinus floor and express chemokine and homing receptors. **a** Representative FACS plots showing the forward and side scatter of naïve (left) and in vitro-activated (right) CD4^+^ T cells as well as the expression of CD62L and CD44. **b** Expression of E- and P-selectin and chemokine receptors on activated CD4^+^ T cells; control CCR7 staining in *Ccr7*^*−*/*−*^ activated CD4^+^ T cells (blue line). **c** Expression of integrin subunits β1 (CD29), β2 (CD18), β7, and αv (CD51) on activated CD4^+^ T cells (red lines); gray shaded areas, isotype control. **d** Representative immuno-fluorescence microscopy of popliteal LN sections 20 and 90 min after i.l. injection of in vitro-activated CD4^+^ T cells and **e** 90 min after i.l. injection of in vivo-activated GFP-OTII cells (green); red, anti-IgD; blue, anti-Lyve-1; scale bars, 100 µm.

To identify potential molecules involved in this homing process, we activated naïve CD4 T cells in vitro by stimulation with IL-2, anti-CD3, and anti-CD28 antibodies. After 3 days of activation, cells showed an increase in size, up-regulation of CD44, and expression of L-selectin (Fig. 2a). These cells also expressed ligands for P- and E-selectin, chemokine receptors such as CCR7, CCR8, and CXCR3 (Fig. 2b), high levels of β1, β2, αv integrins, and low levels of β7 integrin (Fig. 2c). A similar expression pattern was observed in antigen-specific in vivo-activated CD4 T cells (Supplementary Fig. 3). To study their homing behavior, we i.l. injected 4–5 × 10^4^ activated CD4 T cells into the afferent lymph vessel of the popliteal LN. Twenty minutes after delivery, most of the transferred cells were still located in the LN sinus system and only few cells had entered the LN parenchyma, primarily residing within interfollicular areas (Fig. 2d). In contrast, 90 min after i.l. transfer most of the transferred cells had reached the outer paracortical TCZ (Fig. 2d). Thus, in contrast to naïve CD4 T cells^10^, ald T cells are able to enter the LN parenchyma directly through the afferent side SCS floor. In subsequent experiments, we analyzed the intranodal positioning at about 90 min after i.l. delivery, since at this time the majority of transferred T cells had already entered the LN parenchyma while not having reached their final position within the deep TCZ. In addition to in vitro-activated CD4 T cells, we also studied the homing of in vivo-activated CD4 T cells. To this end, we intravenously transferred OTII CD4 T cells into recipients that were immunized with ovalbumin (ova) plus adjuvant. Three days later, activated OTII cells were isolated by flow sorting from spleen and LNs and were then i.l. transferred. Consistently, in vivo-activated, lymph-derived CD4 T cells also entered the LN parenchyma primarily via the SCS floor (Fig. 2e).

### The SCS acts as a 3D sieve for lymph-derived cells

**Fig. 3:**
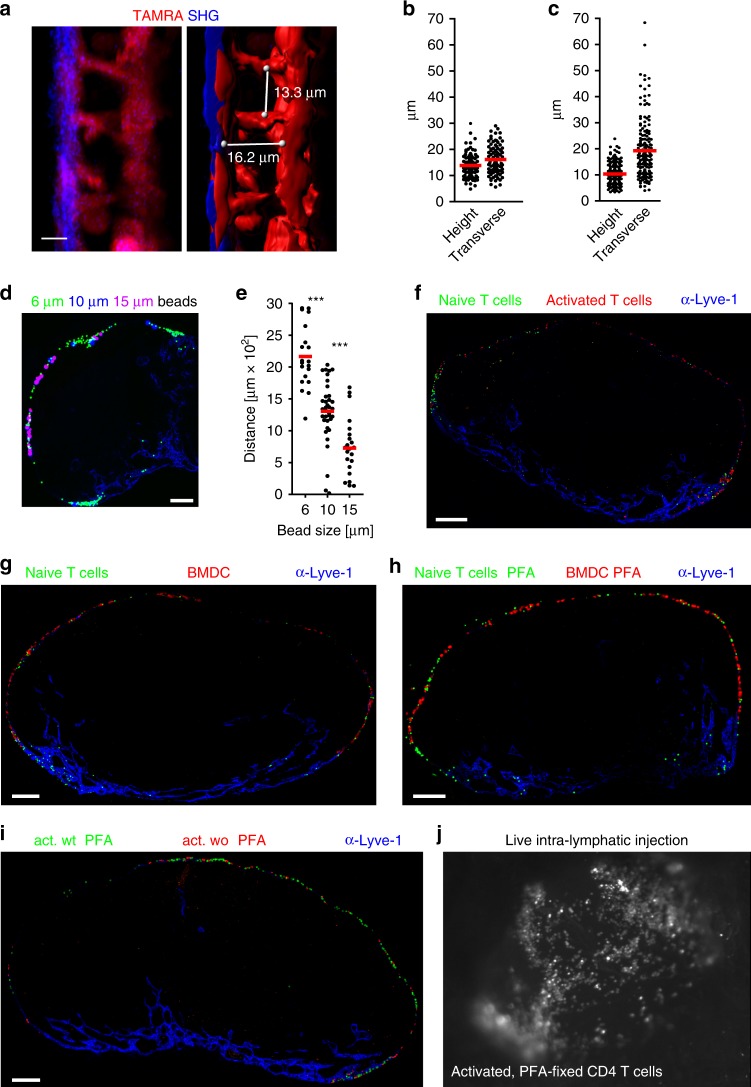
The SCS represents a 3D sieve that acts as a mechanical barrier to arrest lymph-derived activated CD4 T cells. **a** In vivo imaging and three-dimensional reconstruction of an inflamed pop LN SCS after subcutaneous injection of TAMRA. White lines indicate measure points for SCS height and transverse distance between cells/fibers. **b**, **c** Quantitative analysis of SCS heights and transverse distances in resting (**b**) and in inflamed (**c**) pop LNs. **d**, **e** Immuno-fluorescence microscopy (**d**) and quantitative analysis (**e**) of latex bead positioning in popliteal LN SCS after intra-lymphatic injection of fluorescent latex particles (6 µm, green; 10 µm, blue; 15 µm, pink) showing the farthest distance along the SCS between beads of the same size; blue, counterstaining with anti-Lyve-1. **f–i** Immunohistology of popliteal LNs directly after i.l. injection of cells as indicated; **j** snapshot of i.l. live injection of activated PFA-treated CD4 T cells. **a–c** Representative images and analysis of 5–6 LNs analyzed per group in five independent experiments with 1–4 stacks/LN 5–13 measure points per LN stack. **d–e** LNs from seven mice in three independent experiments; **f–i** LN from six mice in two independent experiments; scale bars: **b** 10 µm; **d**, **f**, **g**, **h**, **i** 100 µm, **e** Mann Whitney test, ***p < 0.001; **b**, **c**, **e** red bars, mean.

To gain insights into mechanisms that control the initial arrest of ald T cells within the SCS, we visualized the 3D architecture of the popliteal LN sinus system. We s.c. injected the red fluorescent dye TAMRA and analyzed its distribution in pop LN of untreated mice and mice that received s.c. injection of modified vaccinia Ankara (MVA) into the footpad 3 days earlier resulting in LN swelling at the time of analysis. In vivo two-photon microscopy (2PM) was used to determine the distance between the collagen-rich capsule (revealed by second harmonics generation (SHG); blue) and the TAMRA-stained floor cells, presumably MRCs^3,21^, as well as of collagen-rich fibers spanning between the sinus floor and ceiling, giving rise to an intricate 3D sieve (Fig. 3a). A systematic analysis revealed an average sinus height of 13.9 µm and an average transverse distance between the “strands” of 16.1 µm in the non-inflamed LN (Fig. 3b). Interestingly, in the inflamed LN the height of the sinus was reduced to 10.3 µm while the transverse distance between ceiling–floor spanning strands was increased to 19.3 µm (Fig. 3c). To address whether the 3D architecture of the SCS serves as a size filter for incoming cells, we first i.l. injected latex beads of various sizes (6–15 µm) into afferent lymph vessels. Remarkably, latex beads of all three diameters were retained within the SCS (Fig. 3d). Analyzing the distribution of beads in the SCS by measuring the maximum distance between particles of the same size along the SCS revealed that small particles can easily pass into medullary sinuses while large particles are more frequently retained in the afferent side SCS (Fig. 3e). Consistently, when 15 µm beads were injected into MVA-inflamed LNs, we had to increase injection pressure from 15−25 to 100 kPa in order to deliver beads to the inflamed LN. As a consequence disruption of the SCS was observed, indicating their difficulty to passively distribute along the sinus system (Supplementary Fig. 4). These findings suggest that the SCS provides a structure that allows for both size discrimination and mechanical arrest of incoming cells. To further substantiate this idea, we i.l. injected a mixture of naïve T cells (small) and activated T cells (large) and immediately analyzed their distribution along the LN sinus system. Similar to the beads, naïve T cells could more easily reach the medullary sinuses compared to activated T cells (Fig. 3f). We then injected a mixture of naïve T cells (small) and bone marrow-derived DCs (BMDCs, large). While some of the T cells were passively transported into medullary sinuses, DCs were completely retained in the SCS (Fig. 3g). Interestingly, the same observations were made following injection of naïve T cells and DCs that were both fixed with paraformaldehyde (PFA) prior injection in order to interfere with any active cell adhesion (Fig. 3h). Similarly, activated CD4 T cells either PFA-fixed or left untreated were transported along the circumference of SCS lumen to the same degree (Fig. 3i). Collectively, these data suggest that retention of lymph-derived cells within the SCS is independent of active adhesion molecules. To directly visualize the mechanical arrest of lymph-derived cells within the SCS, we employed epifluorescence microscopic live imaging of the popliteal LN during i.l. transfer. Irrespective of the treatment, delivered cells were observed to passively float into the SCS lumen before coming to an instantaneous stop (Supplementary Movie 1; Fig. 3j). Together, these data strongly suggest that the SCS provides a 3D-sieve structure that acts as a size filter for incoming immune cells, and that the mechanical barrier, rather than active cell adhesion, is a major determinant of cell arrest for lymph-derived cells.

### CD4 T cells move along and through sinus endothelial cells

**Fig. 4:**
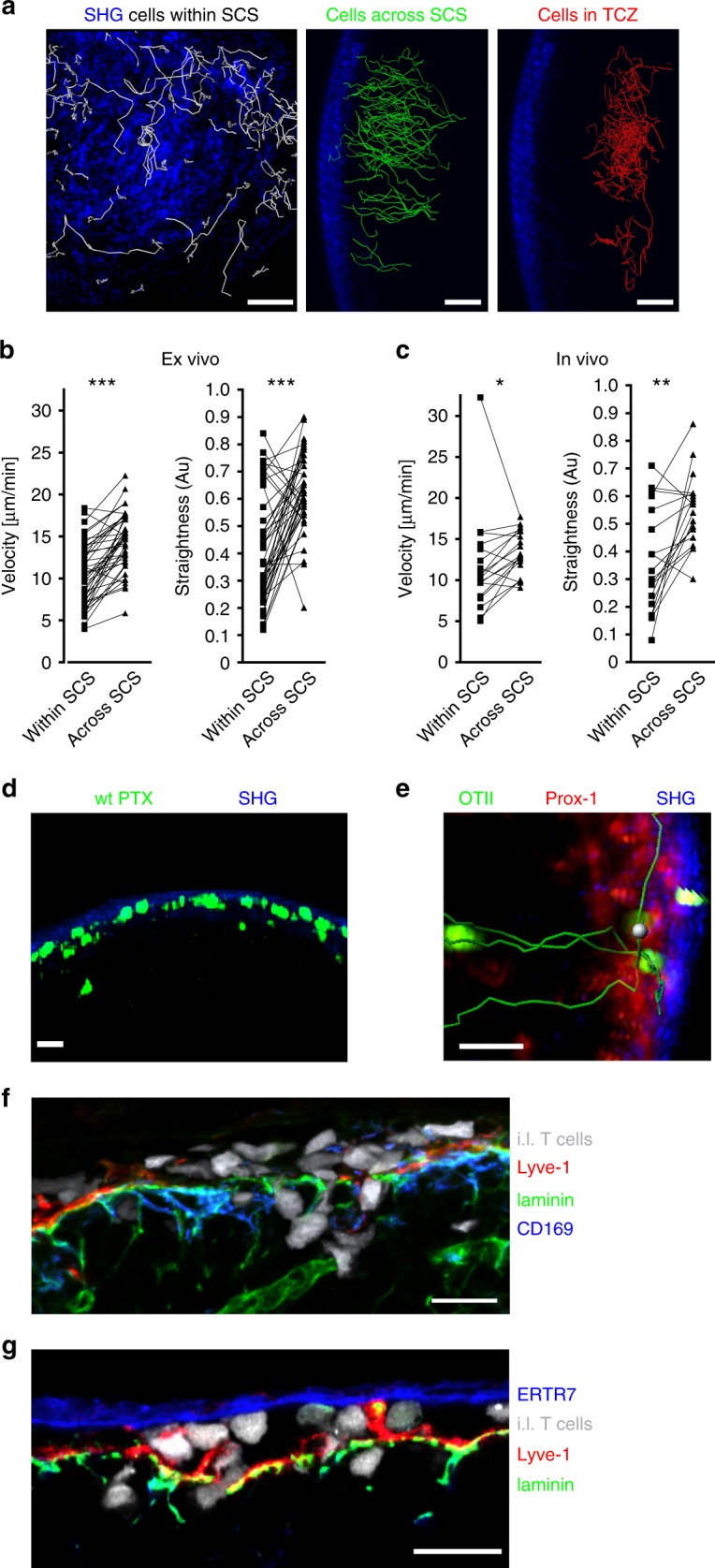
Translocation through the SCS floor but not migration into the lymph node parenchyma occurs independent of chemokine receptors. **a** Ex vivo 2-photon imaging revealed different migration behavior of activated CD4^+^ T cells within the SCS (left, white tracks), directly after crossing the SCS floor (middle, green tracks), or within the LN parenchyma (right, red tracks). Movies start 15 min after i.l. injection of activated CD4^+^ cells into wild-type recipient mice. **b**, **c** Quantitative analysis of migration velocity and straightness of in vivo-activated OTII cells within the SCS or when crossing the SCS imaged ex vivo (**b**) or in vivo (**c**). Data are derived from five to seven movies from three independent experiments; Wilcoxon signed rank test *p < 0.05; **p < 0.01; ***p < 0.001. **d** Ex vivo time-lapse imaging of a popliteal LN after i.l. injection of activated PTX-treated CD4^+^ T cells (green), showing migration activity in the LN SCS. **e** Ex vivo time-lapse imaging of a popliteal LN after i.l. injection of in vivo-activated OTII-GFP cells (green) into a Prox1-mOrange2 recipient mouse showing simultaneous transmigration (tracks in green) of individual cells at the same localization. Prox1 (red) indicates SCS lymphatic endothelial cells. **f**, **g** Confocal images of the SCS showing adoptively transferred cells (white) within the SCS while crossing the SCS floor. Counterstained with antibodies as indicated; scale bars: **a** 50 µm; **d**, **e** 20 µm; **f**, **g** 15 µm.

To further study molecular mechanisms that allow ald T cells to translocate from the SCS into the LN parenchyma, we applied in vivo and ex vivo 2PM. After arrival within the SCS, ald T cells were regularly found to crawl with an average speed of approximately 12 μm/min on endothelial cells of the sinus floor, resembling a 2D random walk pattern (Fig. 4a, white tracks; Fig. 4b–c; Supplementary Movie 2). The process of actually crossing the SCS floor was accompanied by a pronounced increase in T cell speed and directionality, resulting in a highly directional migration towards the TCZ for distances of approximately 50 to 150 µm (Fig. 4a, green tracks; Fig. 4b–c) before homed cells started to adopt a seemingly 3D random walk motility within the deeper LN parenchyma (Fig. 4a, red tracks; Supplementary Movie 2).

Interestingly, activated T cells treated with PTX were still able to crawl on sinus endothelial cells but only occasionally translocated into the LN parenchyma to some extent. However, those PTX-treated cells that had reached the parenchyma completely failed to migrate towards the TCZ and were observed to migrate back into the SCS lumen on several occasions (Fig. 4d; Supplementary Movie 3). Since PTX treatment leads to ADP ribosylation and functional deactivation of all Gα_i_ proteins which are essentially required for the signaling of all chemokine receptors and additional Gα_i_-protein coupled receptors, these findings indicate that chemokines are essentially involved in guiding T cells out of the SCS into the LN parenchyma, but not for basal migration within the sinus. T cells passed through the SCS floor at multiple sites with some cells migrating through the SCS floor at the same location, indicating the existence of preformed structures allowing cell entry (Fig. 4e, Supplementary Movie 4). Indeed, confocal microscopy of the SCS area revealed many pores within the basement membrane and between lymphatic floor endothelial cells and subcapsular macrophages (Fig. 4f–g). Notably, s.c. applied anti-laminin antibodies did not only bind to the SCS basement membrane but also to other laminin-containing structures within the interfollicular area (Fig. 4f–g). Taken together, these observations indicate that, once ald T cells have arrived in the SCS, they actively crawl on endothelial cells of the SCS floor in search of potential entry sites. The crawling movement on endothelial cells occurs independent of chemokine receptor signaling. However, chemokine receptors are essentially required to translocate cells from the SCS towards the TCZ by means of an initially highly directional migration.

### Molecules dispensable for homing via the SCS

**Fig. 5:**
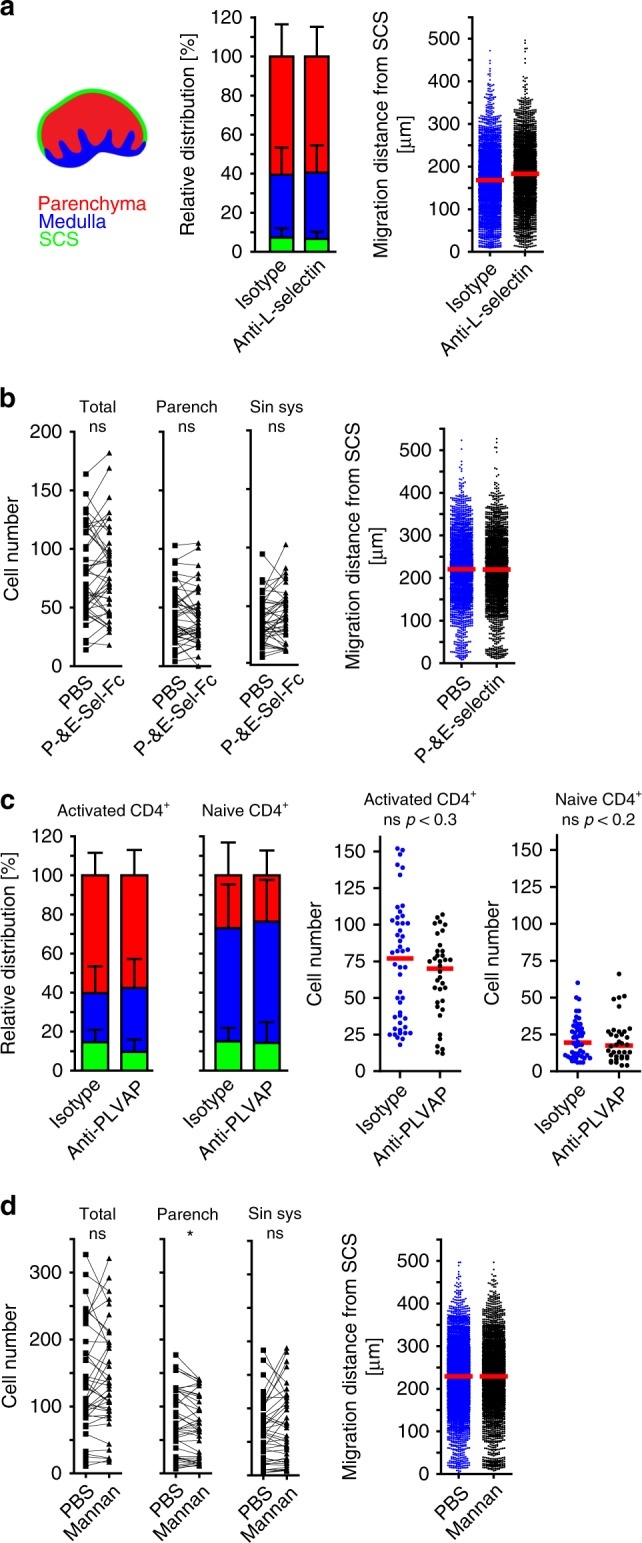
Selectins, PLVAP, and mannan-binding proteins do not contribute to homing of lymph-derived activated CD4 T cells. Quantitative analysis of adoptively transferred cells in popliteal LNs 90 min after i.l. injection of **a**, **b**, **d** 1:1 mixtures of activated CD4^+^ T cell combinations as indicated; **c** activated CD4^+^ T cells into different recipients; **a** relative distribution as schematically depicted and migration distance of activated T cells treated with isotype or anti-L-selectin mAb; **b** absolute distribution and migration distance of activated T cells treated with isotype or anti-P- and E-selectin Ig fusion protein (parench parenchyma, sin. sys sinus system); **c** relative distribution and absolute cell counts of activated T cells injected into recipients treated with isotype or anti-PLVAP mAb; **d** absolute distribution and migration distance of activated T cells treated with PBS or Mannan. Data are derived from three experiments with a total of six mice (**a**), or from two experiments with a total of six (**b**, **c**) and five (**d**) mice; error bars, SD; red bars, median; **b**, **d** Wilcoxon signed rank test *p < 0.05; **c** Mann Whitney test.

Since ald T cells were observed to start crawling on the SCS floor endothelium after the initial mechanical arrest, we aimed to identify molecules that might affect the arrest of T cells in the SCS. We first studied the selectin family known to be important for the homing of immune cells via HEVs to LNs and via activated postcapillary venules to inflamed tissue^2^. We first incubated activated T cells with a neutralizing anti-L-selectin mAb known to completely block lymphocyte homing via HEVs. However, we could not observe any effect of L-selectin blockade on the distribution of i.l. injected T cells (between SCS, medullary sinus, or LN parenchyma) or their subsequent translocation from the SCS towards the TCZ calculated by the shortest distance of transferred cells to the SCS floor (Fig. 5a). Likewise, incubating activated CD4 T cells with both E-selectin-Ig and P-selectin-Ig fusion proteins prior to i.l. transfer had no effect on the relative positioning of these cells—within the sinus system (i.e. within SCS or medullary sinus areas) vs. LN parenchyma—or the migration distance from the SCS (Fig. 5b). The endothelial-expressed adhesion molecule PLVAP has also been suggested to be involved in LN entry of lymphocytes and antigens arriving via afferent lymphatics^16^. Thus, we treated mice with a neutralizing anti-PLVAP mAb (MECA-32). However, anti-PLVAP mAb had no effect on any of the homing parameters studied of neither activated nor naïve CD4 T cells (Fig. 5c). We next tested whether C-type lectins known to be involved in lymphocyte trafficking^22,23^ play a role for T cell homing from the SCS. Blocking C-type lectins on activated T cells by incubation with mannan prior to the adoptive i.l. transfer only slightly affected homing into the parenchyma while no effect on the other homing parameters could be observed (Fig. 5d). Furthermore, our studies neither revealed a role for the hyaluronan receptor CD44 nor the Sphingosine 1 phosphate receptor 1 (S1PR1), in the homing of T cells from the SCS (Supplementary Fig. 5).

### CCR7 is a major homing receptor for activated CD4 T cells

**Fig. 6:**
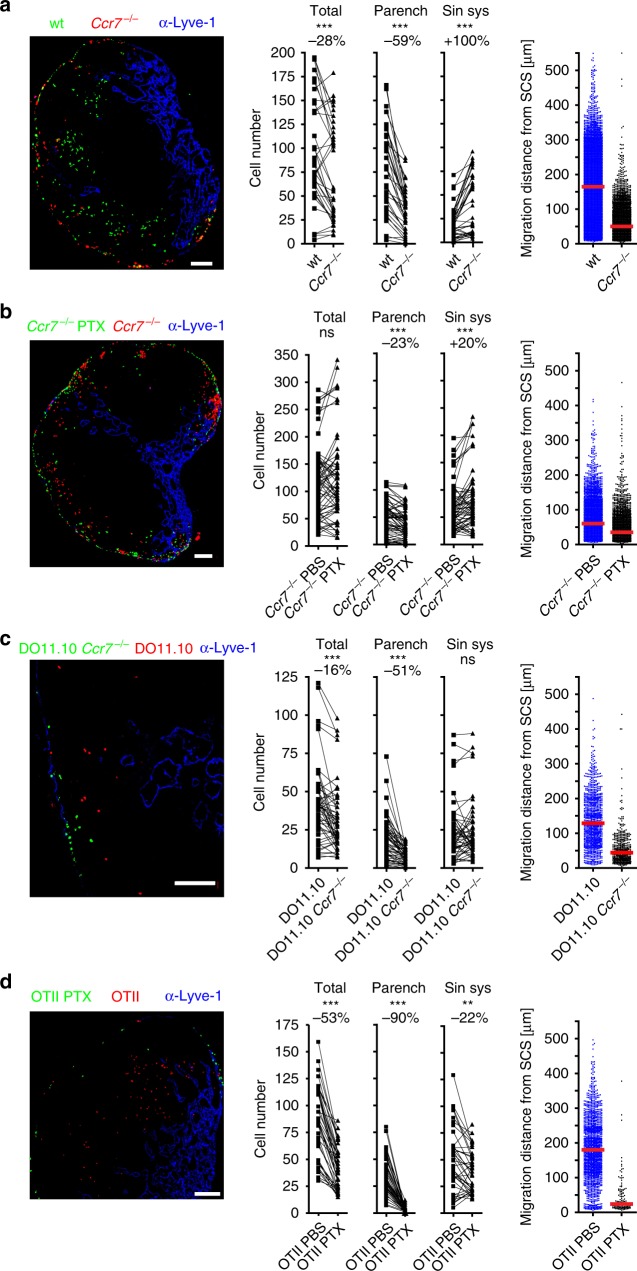
Homing of lymph-derived activated CD4 T cells into LN parenchyma depends on CCR7. Representative images (left) and quantitative analysis (right) of adoptively transferred cells in popliteal LNs 90 min after i.l. injection of 1:1 mixtures of activated CD4^+^ T cell combinations as indicated; **a** in vitro-activated wild-type (wt) and *Ccr7*^*−*/*−*^; **b** in vitro-activated PTX-treated *Ccr7*^*−*/*−*^ and PBS-treated *Ccr7*^*−*/*−*^; **c** in vivo-activated DO11.10 and DO11.10*Ccr7*^*−*/*−*^; **d** in vivo-activated PTX-treated OTII and PBS-treated OTII cells (left; green and red, cells; blue, counterstaining with anti-Lyve-1, scale bars 100 µm). Middle panels, total cell counts in the indicated compartment; dots represent cell counts per LN section; numbers above indicate the percentage of change compared to control cell population; (parench parenchyma; sin. sys sinus system). Right panels, migration distance from the SCS; dots represent cells; red bars, median; ns, not significant; Wilcoxon signed rank test **p < 0.01; ***p < 0.001. Data are derived from four experiments with a total of 12 (**a**) or 7 (c) mice, or from three experiments with a total of eight (**b**) or five (**d**) mice.

We have previously shown that entry of DCs via the SCS floor involves CCR7 (ref. ^10^). We therefore addressed the role of this chemokine receptor in the homing of ald T cells. Ninety minutes after i.l. transfer, significantly lower numbers of *Ccr7*-deficient T cells were found to reside within the target LN (Fig. 6a). Comparing the relative positioning of these cells, we found 59% less *Ccr7*-deficient than wt T cells entering the LN parenchyma, while twice as much *Ccr7*-deficient cells than wt cells were still located in the sinus system (Fig. 6a). Furthermore, on average, wt cells had progressed more than three times further towards the TCZ than those *Ccr7*-deficient cells that had entered the LN parenchyma (Fig. 6a). We previously showed that DCs are impaired in egressing from the SCS into the LN parenchyma in mice deficient for the atypical chemokine receptor (ACKR4)^24^. Since this receptor is expressed on endothelial cells of the SCS and known to scavenge the CCR7 ligands CCL19 and CCL21, we tested whether *Ackr4*-deficiency also affects entry of ald T cells. Our adoptive transfer approach revealed that in *Ackr4*-deficient recipients activated T cells translocate less efficiently towards the TCZ (Supplementary Fig. 6).

To test if chemotactic receptors other than CCR7 are also involved in LN entry and subsequent translocation, we treated in vitro-activated *Ccr7*^*−/−*^ CD4 T cells with PTX prior i.l. transfer. Using gammaretroviral transfer for the expression of GCaMP6, a genetically encoded sensor for Ca^2+^ flux^25^, we confirmed that PTX treatment renders primary T cells insensitive to chemokine-induced Ca^2+^ flux for at least 24 h (Supplementary Fig. 7). Following PTX treatment, the number of *Ccr7*-deficient T cells residing in the sinus system was increased, while the number of cells entering the LN parenchyma was further reduced by an additional 23% (Fig. 6b), indicating that further chemokine receptors or other Gα_i_-coupled receptors contribute to the egress of activated T cells from the SCS. To test whether CCR7 is also involved in regulating homing of cells activated in vivo we i.v. transferred H2-d-restricted DO11.10 CD4 T cells, proficient or deficient for *Ccr7*, into BALB/c recipients that got subsequently immunized with Ova and adjuvant. After 3 days, mice were sacrificed, DO11.10 cells isolated, and subsequently i.l. transferred. These experiments confirmed the importance of CCR7 in guiding activated T cells through the SCS floor and towards the paracortical TCZ (Fig. 6c), while i.l. transfer of in vivo-activated, H2-d-restricted OTII cells treated with PTX reinforced the general importance of chemokine receptors for immigration of T cells via the SCS and their subsequent translocation into the T cell area (Fig. 6d).

### CCR5 and CCR8 contribute to homing of activated CD4 T cells

**Fig. 7:**
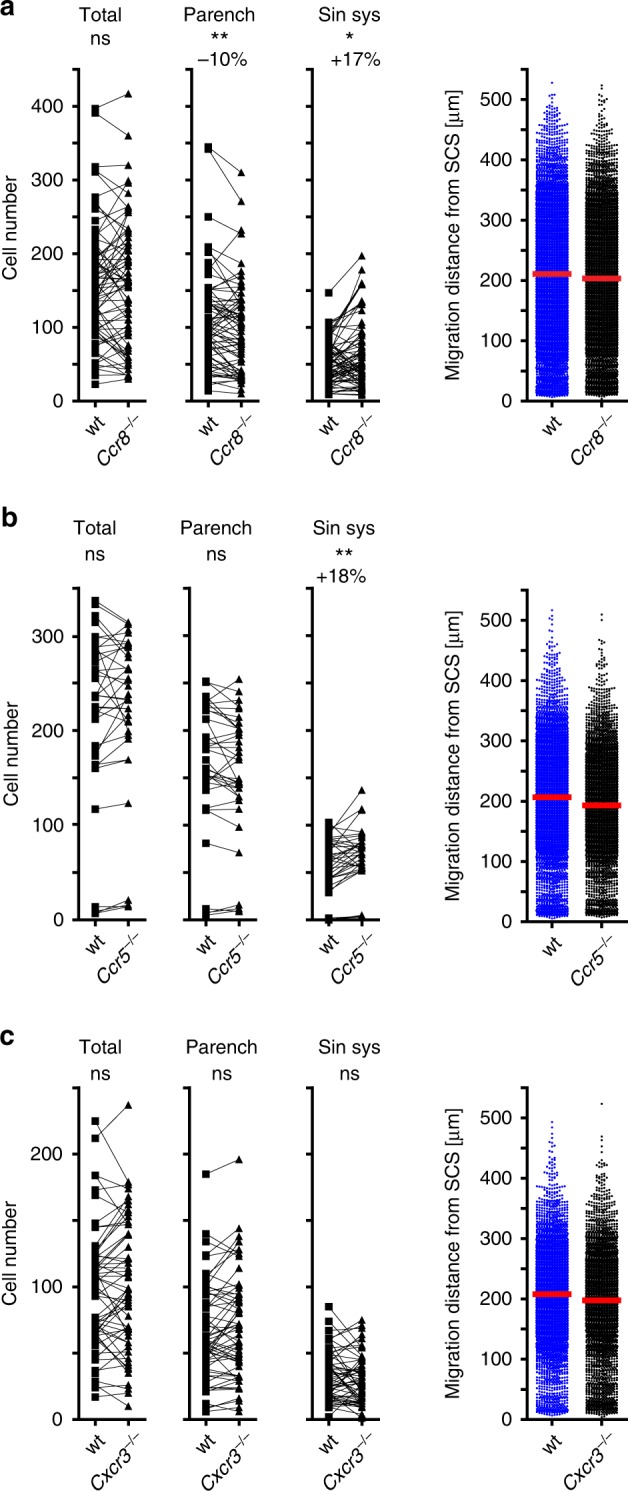
CCR5 and CCR8 but not CXCR3 contribute to homing of lymph-derived activated CD4 T cells to LNs. Quantitative analysis of adoptively transferred cells in popliteal LNs 90 min after i.l. injection of combinations of activated CD4^+^ T cells as indicated; **a** wild type and *Ccr8*^*−*/*−*^; **b** wild type and *Ccr5*^*−*/*−*^; **c** wild type and *Cxcr3*^*−*/*−*^. Left, total cell counts; dots represent cell counts per LN section; numbers above indicate the percentage of change compared to control cell population (parench parenchyma; sin. sys. sinus system). Right, migration distance from the SCS; dots represent the shortest distance of each cell to the SCS; red bars, median; ns, not significant; Wilcoxon signed rank test; *p < 0.05; **p < 0.01. Data are derived from three experiments with a total of nine (**a**) or eight (**c**) mice, or from two experiments with a total of five (**b**) mice.

To address which chemokine receptors besides CCR7 participate in the various steps of LN homing via afferent lymphatics, we compared the homing capacity of activated CD4 T cells proficient or deficient for *Ccr8*, a chemokine receptor known to be involved in LN metastasis^6^. Although lack of *Ccr8* had no effect on the total number of adoptively transferred cells present in the draining LN, the number of *Ccr8*-deficient cells was found to be reduced by 10% in the parenchyma while being increased in the sinus system (Fig. 7a). Notably, *Ccr8*-deficiency had no effect on the average displacement of those cells that had actually entered the LN parenchyma 90 min after transfer. CCR5 is expressed on activated CD4 T cells and adoptive i.l. transfer revealed that, similar to *Ccr8*-deficient T cells, activated T cells deficient for *Ccr5* were more frequently present in the sinus system but not significantly reduced in the LN parenchyma compared to wt cells (Fig. 7b). Again, deficiency for *Ccr5* did neither affect the number of cells that were retained in the LN nor the average translocation distance of immigrated cells (Fig. 7b). Conversely, *Cxcr3-*deficiency had any effect on the homing parameters analyzed in this study (Fig. 7c). Collectively, these data indicate that CCR7 is the chemokine receptor most relevant for guiding activated T cells from the SCS directly via the SCS floor into the LN parenchyma, and that other chemokine receptors, such as CCR5 and CCR8, contribute to this process. Interestingly, in recipients that received s.c. injection of MVA into the foodpad earlier homing of ald T cells was also primarily mediated by CCR7 (Supplementary Fig. 8).

### Integrins help T cell homing and intranodal translocation

**Fig. 8:**
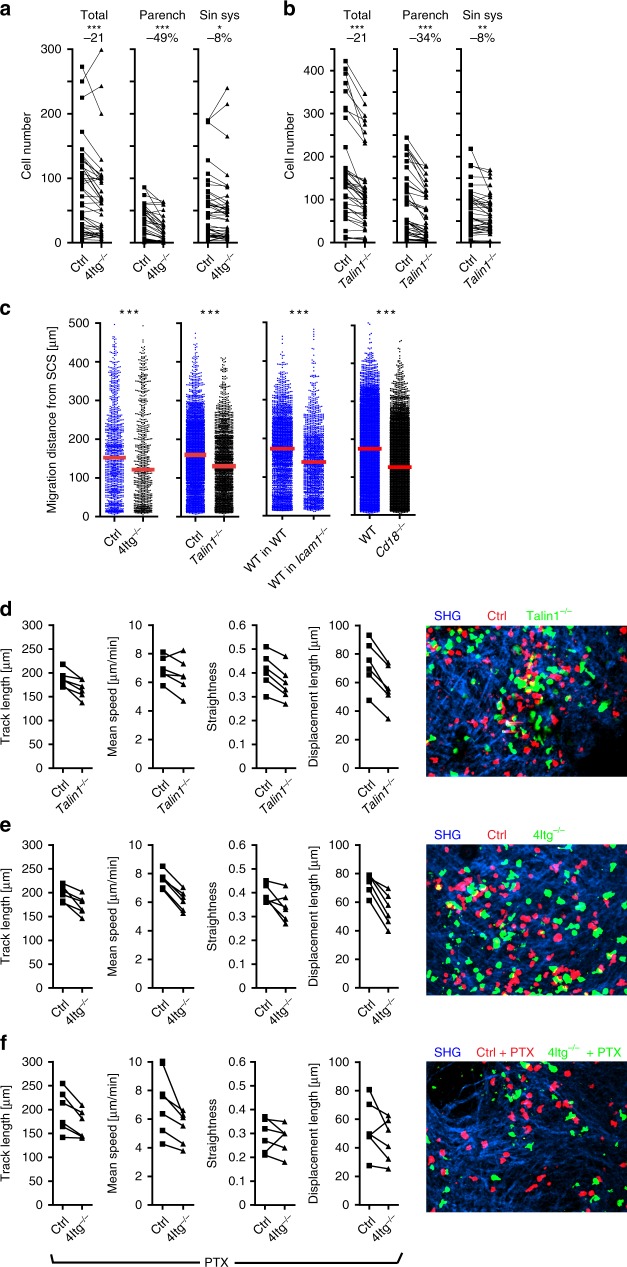
Integrins and Talin1 contribute to the homing of CD4 T cells into the LN after i.l. injection. Quantitative analysis of adoptively transferred cells in popliteal LNs 90 min after i.l. injection of combinations of activated CD4^+^ T as indicated: **a** wild type and *4Itg*^*−/−*^; **b** wild type and *Talin-1*^*−*/*−*^. **a**, **b** Left, total cell counts; dots represent cell counts per LN section; numbers above indicate the percentage of change compared to wild type (parench parenchyma, sin. sys. sinus system). **c** Migration distance of adoptively transferred cells from the SCS as indicated; dots represent cells; red bars, median; ns, not significant; *p < 0.05; **p < 0.01; ***p < 0.001 (paired *t*-test). Analysis of intranodal CD4^+^ T cell migration (left) and representative movies (right) (**d**–**f**) based on ex vivo time-lapse imaging of popliteal LNs after i.l. injection of activated wild type and *Talin1*^*−*/*−*^ (**d**); wild type and 4*Itg*^*−*/*−*^ (**e**), or PTX-treated wild type and 4*Itg*^*−*/*−*^ (f) CD4^+^ T cells. Data calculated based on all cell tracks present within selected regions using automated tracking with manual correction. Dots represent the mean of one cell population per movie. ns not significant; **a**, **b** Wilcoxon signed rank test; **c** Mann Whitney test *p < 0.05; **p < 0.01, and ***p < 0.001 (paired *t*-test). Data are derived from three experiments with a total of eight (**a**, **c**) or four (**d–f**) mice, or from two experiments with a total of six mice (**b**, **c**). Scale bars 50 µm.

To address the role of integrins in the homing of ald T cells we used lentiviral-based CRISPR/Cas9 technology to simultaneously knockout *Itgb1, Itgb2, Itgb7*, and *Itgav* genes encoding for β1 (CD29), β2 (CD18), β7, and αv (CD51) integrins, respectively (Supplementary Fig. 9). Immune cells lacking these 4 integrin (4Itg^*−*/*−*^) genes additionally sorted for those not expressing any remnant molecules do not express any of the known alpha-beta integrin chain pairs (Supplementary Fig. 9e). Following i.l. transfer and immunohistological analysis, the total number of adoptively transferred 4Itg^*−*/*−*^ T cells present in the draining LN was reduced by 21% while the number of cells in the LN parenchyma was reduced by 49% indicating that integrins substantially contribute to the translocation of ald T cells from the SCS into the LN parenchyma (Fig. 8a). To further confirm these results, we also knocked out *Talin1* that encodes an adaptor molecule indispensable for integrin function (Supplementary Fig. 10). Indeed, adoptive transfers revealed that the total number of adoptively transferred *Talin1*^*−/−*^ cells within targeted LN was reduced by 21%, while those having migrated into the LN parenchyma were reduced by 34% (Fig. 8b).

Interestingly, the median migration distance of those cells that entered the LN parenchyma was reduced by 20% in 4Itg^*−*/*−*^ T cells (wt: 153 µm, 4Itg^*−*/*−*^: 121 µm) and by 17% in *Talin1*^*−/−*^ T cells (wt: 156 µm, *Talin1*^*−*/*−*^: 130 µm; Fig. 8c). A similar decrease in translocation was observed for wt cells transferred into *Icam1*^*−/−*^ recipients and for activated CD4 T cells from *Itgb2*-deficient donors following their transfer into wt recipients (Fig. 8c). In contrast to findings reported earlier for DCs^26^, these data suggest that integrins contribute to the translocation of ald T cells from the SCS into the LN parenchyma and their subsequent migration towards the TCZ. The latter effect is mediated by the interaction of T cell-expressed β2 integrin (CD18) and ICAM1 expressed on cells or structural components of the LN environment. To further assess the role of integrins and talin1 on migration characteristics of activated T cells we applied 2PM following i.l. transfer. Compared to wt cells, both *Talin1*^*−/−*^ as well as 4Itg^*−*/*−*^ cells showed a reduction in track length, mean track speeds, track straightness, as well as displacement length (Fig. 8d–e; Supplementary Movies 5 and 6), confirming results obtained by immunohistology.

To characterize migration of ald T cells in the absence of both integrin as well as chemokine receptor signaling, we compared wt and 4Itg^*−*/*−*^ T cells both treated with PTX before i.l. transfer. T cells deficient for all integrins and blocked chemokine receptor signaling showed reduced track straightness and displacement length but still migrated with approximately 6 µm/min compared to approx. 8 µm/min of non-treated wt cells (Fig. 8f; Supplementary Movie 7). Although impaired to some degree, these data show that T cells perform random migration within the SCS in the absence of chemokine as well as integrin signaling.

## Discussion

It is common knowledge that lymphocytes can enter LNs either from the blood via specialized HEVs or from afferent lymph draining into the SCS^1,8,13,17,27^. Whereas LN homing via HEVs has been explicitly studied, little is known about molecular mechanisms and pathways that enable afferent lymph-derived immune cells to actually enter the LN parenchyma. The combination of i.l. immune cell transfer, photo-conversion, and high-resolution live imaging revealed in the present study both fundamental differences but also compelling similarities regarding the various steps of T cell homing via HEVs and afferent lymphatics.

To better understand homing of lymph-derived T cells, we first developed an approach based on the i.v. transfer of CD4 T cells derived from Dendra2-H2B reporter mice crossed to OTII mice combined with the subsequent activation and photo-conversion through intact skin. Analysis revealed efficient homing of CD4 T cells primed in pop LNs to downstream LNs. Following activation by DCs, CD4 T cells are known to egress from draining LNs after the first rounds of cell divisions before gaining full effector functions and distribute to other secondary lymphoid organs. It has been reported that this distribution can occur systemically via blood^28–30^, but the present study implies that activated cells that leave the LN from the lymphatics travel through the chain of downstream LNs before returning to the blood circulation. However, further studies are needed to elucidate the biological significance of this migratory behavior. Moreover, the reasons why T cells egress prematurely are not fully understood. Competition for cytokines in the primary LN required for proliferation and differentiation would allow early T cell disseminators to occupy empty niches in downstream LNs to mount a larger total T cell response. Furthermore, early CD4 T cell disseminators might already secrete cytokines and help to establish a protective immune response in downstream LNs to prevent pathogen spread^31^.

Homing via HEVs is best characterized for naïve T and B cells comprising a multistep adhesion cascade including rolling, vascular sticking, crawling, and trans-endothelial migration^1^. Rolling of lymphocytes along HEV walls occurs via binding of lymphocyte-expressed L-selectin to 6-sulpho sialyl Lewis X motifs on HEV sialomucins^1,32,33^. Rolling lymphocytes are then activated by chemokines such as CCL19 and CCL21 found on the luminal site of HEVs that, after binding to their cognate chemokine receptor (CCR7) and together with shear forces mediated by the flow of blood, induce conformational changes in αLβ2 integrin (LFA-1; CD11a/CD18) allowing firm binding (sticking) to ICAM1 and ICAM2 (refs. ^13,27,34^). After firm arrest is achieved, lymphocytes have been described to crawl for some minutes in a non-directional manner on the inner surface of HEVs and then rapidly transmigrate across these endothelial cells via so called exit ramps most likely established by fibroblastic reticular cells^35–38^. Lymphocytes deficient in *Igtal* or *Ccr7* or treated with neutralizing anti-L-selectin mAb are largely impaired in their capacity to home to LNs via HEVs^12,39–41^. On the other hand, effector T cells express ligands for E- and P-selectin that facilitate their rolling on inflamed endothelium, thus allowing their subsequent migration to peripheral sites of inflammation and infection^33,42^. Importantly, the activated CD4 T cells used in the present study were L-selectin^+^CCR7^+^ and expressed several integrin chains as well as ligands for P- and E-selectin. We therefore tested whether selectins, integrins, and chemokine receptors were involved in the migration process from the SCS into the parenchyma.

We applied adoptive i.l. injection, a method previously established in our group^10^, to transfer CD4 T cells activated in vitro or in vivo for 3 days. We chose this duration of T cell activation since it is known that T cells, activated in vivo by DCs presenting cognate antigen, start to leave the LN via efferent lymphatics around this time after initial antigen contact^43–45^.

Several lines of evidence suggest that the initial arrest of incoming immune cells within the SCS is mediated to a large degree by the mechanical barrier function of the SCS acting as a 3D sieve rather than requiring the action of adhesion molecules. (i) The size of i.l. transferred beads dictates the distance they passively travel within the SCS. (ii) i.l. delivered untreated or PFA-fixed cells of the same size distribute equally within the SCS. (iii) Lack of visible differences in the kinetics of how live cells and fixed cells are stopped within the SCS. We are aware that these findings do not formally exclude the potential involvement of adhesion molecules during the very early arrest step of incoming cells; however, they provide ample evidence that the mechanical barrier of the 3D-sieve structure of the SCS likely dominates this process. The presence of such a filtering system allows for the mechanical arrest of incoming activated T cells and DCs to the primary draining LN and facilitates the initial local confinement of an adaptive immune response to the primary draining node^10^. This model does not exclude the possibility that adhesion molecules, such as integrins, contribute to efficient LN homing, for example, by increasing the dwell time of incoming cells within the SCS. After the initial arrest, some cells reorganize their cytoskeleton and start to crawl on the SCS. While migration within the SCS was largely unimpaired in PTX-treated cells, these cells only occasionally made it into the interfollicular area and were constantly excluded from the deep TCZ. Hence, the chemokine system seems to largely increase the chance of transmigration by “luring” cells through the SCS floor towards the LN parenchyma. In addition to chemokine receptors, PTX treatment could led to the inhibition of other receptors that are linked to cell migration which also couple to Gα_i_-proteins such as S1PR1 (ref. ^46^). However, neither blockade of S1PR1 by pharmacological inhibition with FTY720 nor by genetic deletion of the receptor did not show any effect on CD4 T cell homing making it unlikely that S1P receptors have major impact on the migration of CD4 cells from the SCS lumen into the LN parenchyma.

These findings are in line with a recent study reporting that innate-like lymphocytes that had been guided by CCR6 within the LN parenchyma towards the SCS area use S1P1 at a subsequent step to actively migrate from the LN parenchyma into the SCS lumen^47^. In the present study we identified CCR7 as the major chemokine receptor that guides ald CD4 T cells away from the SCS towards the deep TCZ which is consistent with a previously described CCL21 gradient present within interfollicular areas pointing from the SCS (low) towards the TCZ (high)^24^. An earlier study addressed the role of integrins for LN homing of s.c. applied DCs^26^. In that study, DCs, derived from mouse fetal liver hematopoietic cells deficient for β1, β2, β7, and αv integrins, were not impaired in homing to and appropriately positioning within the draining LN. Since mice deficient for all eight alleles of the above-mentioned integrins are non-viable, the role of integrins on immune cells other than DCs has not been addressed so far. We therefore used a lentiviral CRISPR/Cas9 approach to create a population of activated T cells not expressing any of the known integrins. The i.l. transfer of these cells or of T cells with CRISPR/Cas9-mediated deletion of *Talin1* revealed that integrins contribute to at least two steps of ald T cell homing. In the absence of integrins or talin1, less T cells entered the LN parenchyma and translocated slightly less efficient towards the TCZ due to impaired speed and directionality. Since both T cells deficient for β2 integrin (CD18) and an environment deficient for *Icam-1* resulted in reduced translocation towards the TCZ, it seems most likely that the interaction of αLβ2 integrin (LFA-1) with ICAM-1 enhances the amoeboid migration of T cells. In a recent publication, the group of M. Sixt addressed the role of LFA-1 in intranodal migration of naïve T cells^48^. They observed that *Itgal*-deficient T cells migrated with an approximately 20% reduced velocity compared to wt T cells and put forward the model that T cells migrate in a continuous sliding rather than a caterpillar-like manner. In that model, LFA-1 acts as a frictional clutch with the environment without adhesively confining the cell to its ICAM-expressing environment. Data of the present study support and extend this model. We show that T cells without any integrins are only moderately affected in their migration dynamics within the LN parenchyma as well as in the SCS lumen. Moreover, the additional complete blockage of Gαi signaling did not substantially interfere with migration dynamics within those two compartments.

Activated CD4 T cells did not alter the positioning of co-injected naïve T cells which localized to the peripheral medullary sinuses. This finding differs from our previous observations were some of the naïve T cells co-injected with DCs were actively retained within the SCS (10). The reason for this difference remains elusive but it seems possible that DCs—but not activated CD4 T cells—induce morphological alterations within the SCS floor that also allow naïve T cells to enter the LN parenchyma. One important issue that needs to be addressed in future studies is the route of entry of lymph-derived memory T cells, a relevant cell population in afferent lymphatics with a fast pattern of reactivation which may rely on different recirculation strategies.

In an earlier study, Rantakari et al. suggested a role for SCS floor endothelial cell-expressed PLVAP—that contributes to fibrils in trans-endothelial channels—in the homing of lymph-derived T cells^16^. The authors s.c. injected splenocytes and found increased numbers of transferred cells in the popliteal LN in *Plvap*^*−/−*^ mice, while reduced T cell counts were observed in recipients treated with the anti-PLVAP mAb MECA-32. Based on their observations, the authors suggest that PLVAP is involved in the migration of lymph-derived T cells into the LN parenchyma^16^. In the present study we i.l. injected both naïve as well as activated CD4 T cells, but the MECA-32 mAb had no effect on LN parenchymal homing. Reasons for this discrepancy are currently unclear but it seems possible that systemic treatment of the recipient mice with the anti-PLVAP mAb might have interfered with the entry of s.c.-injected T cells into terminal lymphatics, a step that was bypassed in the present study by directly delivering the cells into the afferent lymphatic vessel lumen via cannulation. It seems plausible as well that the time span following cell transfer after which homing was analyzed (4 h vs. 1.5 h in the present study) had an effect on the outcome of the experiments. Alternatively, considering that on average less than 0.1% of s.c. applied splenocytes reach draining LNs, it seems also possible that the few cells that successfully homed into the LN in that setup were actually selected based on their ability to utilize PLVAP. In contrast, the present study reveals that the basement membrane of the SCS floor contains numerous pores through which ald T cells translocate from the SCS lumen into the LN parenchyma. Gaps in the SCS floor (0.1–1 µm) have been identified by electron microscopy^4,49,50^ and were shown to allow rapid transfer of large soluble antigens directly into follicles where they are taken up by B cells carrying cognate B cell receptors^51^. The present study also revealed that s.c. applied anti-laminin antibodies not only rapidly bind to the SCS basement membrane but also to further laminin-containing structures deep in the interfollicular area presumably by leaving the SCS through the gaps in the floor. Although not formally addressed in the present study, it seems likely that ald T cells also use these gaps to translocate from the SCS into the LN parenchyma.

In summary, we identify a multistep mechanism of LN homing of afferent lymph-derived lymphocytes (Supplementary Fig. 11). In contrast to homing of blood-borne lymphocytes via HEVs, the initial arrest of lymph-derived activated T cells in the SCS does not rely on adhesion molecules but is primarily achieved by the 3D-sieve structure of the SCS itself acting as a mechanical barrier. Arrested cells start to crawl back and forth on the SCS floor endothelium until they encounter preformed entry ramps that allow their translocation out of the sinus lumen. Cell crawling on the SCS is highly active even in the combined absence of any integrin as well as G_αi_ signaling. However, guidance into LN parenchyma entirely depends on chemokine receptor signaling and is reduced in the absence of integrins. Once within the LN parenchyma, ald T cells perform a chemokine driven, highly directional migration towards the TCZ for distances of some 100 µm with CCR7 playing a major role for ald T cells.

## Methods

### Animals

Mice were bred at the Central Animal Facility at Hannover Medical School under specific pathogen-free conditions or purchased from Charles River (Sulzfeld, Germany). The following mouse strains were used: C57BL/6, BALB/c, F1-cross between mice expressing GFP under the beta-actin promoter CByJ.B6-Tg(CAG-EGFP)1Osb/J (Stock No: 007075)^52^ and T cell receptor transgenic OTII mice B6Cg-Tg(TcraTcrb)425Cbn/J (Stock No: 004194)^53^ (OTII-GFP), B6.Cg-Commd10Tg(Vav1-icre)A2Kio/J (Stock No: 008610)^54^, B6.129 P2(C)-Ccr7tm1Rfor/J (Stock No: 006621)^12^ (CCR7^*−*/*−*^), B6.129P2-Ccr5tm1Kuz/J (Stock No: 005427)^55^ (CCR5^*−*/*−*^), BALB/c-Tg(DO11.10)10Loh/J (Stock No: 003303)^56^ (DO11.10), BALB/c-Tg(DO11.10)10Loh/J × BALB/c-Ccr7tm1Rfor (DO11.10CCR7^*−*/*−*^), B6.129P2-Cxcr3tm1Raks^57^ (CXCR3^*−*/*−*^), B6.129P-CCR8tm1Lira^58^ (CCR8^*−*/*−*^), B6.129 prox1-mOrange2-pA-BAC^59^ (Prox1-mOrange2), B6.129S4-Icam1tm1Jcgr/J (Stock No: 002867)^60^ (ICAM1^*−*/*−*^), B6J.129(Cg)-Gt(ROSA)26Sortm1.1(CAG-cas9*,-EGFP)Fezh/J (Stock No: 026179)^61^, B6.129S6-Ackr4tm1.1Rjbn^62^ (*Ackr4*^*−/−*^). Mice expressing a stop-flox cassette in front of H2B-Dendra2 within the *Rosa26* locus were generated by Cyagen. Mouse genomic fragments containing homology arms were amplified from BAC clone by using high fidelity Taq, and were sequencially assembled into a targeting vector together with recombination sites and selection markers. The linearized vector was subsequently delivered into embryonic stem (ES) cells from C57BL/6 mice via electroporation. Correctly targeted ES clones were confirmed via Southern blotting before blastocyst microinjection (C57BL/6) and chimera production. Mice used for experiments were usually 6- to 12-week-old. Females and males were used. Mice were euthanized by CO_2_ inhalation and cervical dislocation. Due to the large number of different mouse strains used in this study we could not use littermates as wild-type controls. All experiments were conducted in accordance with the local animal welfare regulations reviewed by the institutional review board and the Niedersächsisches Landesamt für Verbraucherschutz und Lebensmittelsicherheit (LAVES).

### Antibodies and reagents

Antibodies and reagents were as follows: anti-Human/Mouse CD44 eFluor450 (IM7) (# 48-0441; at a 1/200 dilution), anti-Human/Mouse CD44-Biotin (IM7) (# 13-0441; 1/200), eFluor 660-conjugated anti-LYVE-1 (ALY7) (# 50-0443; 1/50), Cell Proliferation Dye eFluor670 (# 65-0840) and eFluor450 (# 65-0842), PE anti-mouse CD197 (CCR7, 4B12) (# 12-1971; 1/20), APC anti-mouse CD183 (CXCR3-173) (# 17-1831; 1/200), Biotin Anti-mouse CD18 (M18/2) (# 13-0181; 1/100), Biotin Anti-mouse CD29 (HMb1-1) (# 13-0291; 1/400), Biotin Anti-mouse Integrin β7 (FIB504) (# 13-5867; 1/400), Biotin Anti-mouse CD51 (RMV-7) (# 13-0512; 1/50), Biotin Armenian Hamster IgG Isotype control (# 13-4888; 1/50), Streptavidin-Cy5 (# 17-4317; 1/200) (all from eBioscience); Alexa Fluor 647 goat anti-mouse IgG (Thermo Fisher Scientific) (# A-21235; 1/1000); mouse monoclonal Anti-Talin1 (8D4) (# ab157808; 1/200) and Mouse IgG1 isotype control (15-6E10A7) (# ab170190; 1/200) (both from Abcam); PerCP anti-mouse CD4 (RM4-5) (# 100537; 1/200), APC anti-mouse CD62L (MEL-14) (# 104411; 1/200), PE/Cy7 anti-mouse TCR β chain (H57-597) (# 109221; 1/200), Alexa Fluor 594 anti-mouse CD169 (siglec-1) (3D6.112) (# 142416; 1/100), all from Biolegend; anti-mouse Panendothelial Cell Antigen (PLVAP, MECA-32) (# 550563; 1/50) and recombinant mouse P-Selectin-IgG fusion protein (#555294) (BD), recombinant mouse E-Selectin/Fc Chimera (#575-ES-100) (R&D Systems); anti-mouse IgD-Cy3 (HB250) (1/100), anti-mouse CD4-Cy3 (1/100), and anti-mouse CD4-Cy5 (1/50) (both RmCD4) were prepared in house; biotin anti-mouse DO11.10 TCR (KJ126) (# MM7515) from Caltag laboratories, rat anti-mouse ER-TR7 (# MCA2402, 1/50) from Bio Rad, rabbit anti-mouse laminin (# LSL-LB-1013, 1/50) from Cosmo Bio, rabbit anti-mouse LYVE-1 (# DP3513, 1/50) from Acris, Alexa Fluor 647-labeled CCL1 (# CAF-07, 1/200) from Almac, FITC goat anti-rabbit IgG (# 111-095-003), Alexa Fluor 647 mouse-anti-rat IgG Fc Fragment specific (# 212-605-104), R-phycoerythrin-conjugated AffiniPure goat anti-human IgG, Fcγ Fragment specific (# 109-115-098) (all Jackson Immuno Research), propidium iodide (Fluka), DAPI (4,6-diamidino-2-phenylindole), Pertussis toxin from *Bordetella pertussis*, Freund’s adjuvant complete, PFA, ovalbumin (Albumin from chicken egg white), mannan from *Saccharomyces cerevisiae* (all from Sigma Aldrich), 6 µm yellow-green fluorescent polystyrene latex microspheres (Polysciences), 10 µm crimson fluorescent FluoSpheres polystryrene microspheres and 15 µm red fluorescent FluoSpheres polystryrene microspheres, TAMRA (*N*,*N*,*N*′,N′-tetramethyl-5-(and-6-)-carboxyrhodamine succinimidyl ester), streptavidin Alexa Fluor 488 conjugated (all Thermo Fisher Scientific), IgG2a isotype control (provided by E. Kremmer, München). Talin-1 intracellular staining was performed as recommended by the manufacturer.

### Vector construction and viral particle production

Lentiviral vector expressing sgRNA was generated as previously described^63^. In this study vectors expressing dTomato (pLKO5.hU6.sgRNA.BsmBI-Stuffer.dTomato.PRE), Cerulean (pLKO5.hU6.sgRNA.BsmBI-Stuffer.Cerulean.PRE), or eYFP (pLKO5.hU6.sgRNA.BsmBI-Stuffer.eYFP.PRE) fluorescent markers were used. To construct gRNA expressing vectors, 20 bp target sequences were designed using http://crispr.mit.edu/ and https://crispr.cos.uni-heidelberg.de/ online tools^64^ and cloned behind human U6 promoter by cutting the vector with BsmBI followed by ligation. The targeted sequences (20 bp target) used in this study include *Tln1*, GATAATGCCCTACGAGCCGT (sequence kindly provided by Prof. Michael Sixt, Institute of Science and Technology Austria); *Cd44*, CATGGAATACACCTGCGTAG; *Itgb1*, GAGGAATGTAACACGACTGC; *Itgb2*, GTGACTTTCCGGCGGGCCAA; *Itgb7*, CGTGACGCGGATCCGCTGCG; *Itgav*, TCATGGACCGAGGTTCCGAT. For cloning we used the following primer pairs: Talin1 5′-CACCGATAATGCCCTACGAGCCGT-3′ and 5′-AAACACGGCTCGTAGGGCATTATC-3′; Cd44 5′-CACCGCATGGAATACACCTGCGTAG-3′ and 5´-AAACCTACGCAGGTGTATTCCATGC-3′; Itgb1 5′-CACCGAGGAATGTAACACGACTGC-3′ and 5′-AAACGCAGTCGTGTTACATTCCTC-3′; Itgb2 5′-CACCGTGACTTTCCGGCGGGCCAA-3′ and 5′-AAACTTGGCCCGCCGGAAAGTCAC-3′; Itgb7 5′-CACCGCGTGACGCGGATCCGCTGCG-3′ and 5′-AAACCGCAGCGGATCCGCGTCACGC-3′; Itgav 5′-CACCGTCATGGACCGAGGTTCCGAT-3′ and 5′-AAACATCGGAACCTCGGTCCATGAC-3′. For construction of vectors targeting two different genes, 368 bp DNA fragments containing hU6 promoter and target sequences against *Itgav* or *Itgb7* were amplified by PCR and inserted into *Eco*RI site of vectors containing target sequences against *Itgb1* and *Itgb2*, respectively.

Lentiviral particles were produced by transient transfection of HEK-293 T cells using a calcium phosphate transfection kit (Sigma Aldrich). One day before, 5 × 10^6^ 293 T cells were seeded per 10-cm dish. Lentiviral supernatants were produced by simultaneously co-transfecting packaging cells with 5 μg sgRNA-expressing vector, 5 μg pRSV-Rev (kindly provided by T. Hope, Northwestern University, Chicago, IL, USA), 12 μg pcDNA3.gp.4xCTE (HIV-1 Gag-Pol)^65^, and 3 μg K73 pEcoEnv-IRES-puro^66^. Transfection medium was supplemented with 20 mM HEPES and 25 μM chloroquine (both Sigma Aldrich)). Supernatants were harvested 24 and 48 h after transfection, filtered through a 0.22-μm pore-size filter (Carl Roth, Karlsruhe, Germany) and concentrated (100×) by ultracentrifugation (16–18 h, 13,238 × *g*, 4 °C) (SW32Ti rotor; Beckman Coulter GmbH, Krefeld, Germany). Viral pellets were re-suspended in DMEM and stored in aliquots at −80 °C until usage.

### Cas9 ribonulcleoprotein preparation and transfection

Sequences of *S1pr1* crRNAs were selected from pre-designed crRNAs from Integated DNA Technologies Inc. (IDT; https://eu.idtdna.com/pages/products/crispr-genome-editing/alt-r-crispr-cas9-system) website. In total, three anti-*S1pr1* crRNA (AB 5′-GGTGTCCACTAGCATCCCGG-3′, AC 5′-CGGCCCATGTACTATTTCAT-3′ and AD 5′-GCGGCTTCGAGTCCTGACCA-3′) were individually mixed with unmodified tracrRNA (all from IDT) in 1:1 molar (210 pmol) ratio, heated at 95 °C for 5 min, and slowly cooled to room temperature to assemble crRNA:tracrRNA complexes. Afterwards, three crRNA:tracrRNA complexes were pooled together before Cas9 NLS protein from *S. pyogenes* (IDT) was added in 3:1 molar ratio (210 pmol of Cas9 to total of 630 pmol of crRNA:tracrRNA complexes) and left at room temperature for 10–20 min before used for transfection. As a control, we used negative control crRNA (IDT) that has no specificity to mouse, rat, or human genome to prepare Cas9 RNPs.

Prepared Cas9 RNPs were used for nucleofection using Primary Cell 4D-Nucleofector X kit L (Lonza) and a 4D nulceofector X and Core units (both Lonza). Briefly, for each nucleofection 2 ml of complete T cell medium was pre-warmed in a cell incubator. 1–5 × 10^6^ activated mouse CD4^+^ T cells were resuspendend in 100 µl of primary cell nucleofection solution and added to the cuvette pre-loaded with total of 10 µl RNPs. Cells were immediately electroporated using pulse program DN-100. Afterwards, pre-warmed complete T cell medium was used to transfer nucleofected CD4^+^ T cells in six-well plates. Nucleofection was done twice, on day 2 and day 4 of cell culture protocol as described above.

To confirm gene-knockout efficacy, Cas9 RNP target sites were amplified by PCR from bulk DNA isolated from nucleofected cells, purified, and sent for Sanger sequencing as described earlier^67^ using following primer pairs: S1PR1-AB 5′-GCT GGGCATTTTCCCTGATTC-3′ and 5′-GCAAGGAGGCTGAAGACTGA-3′; S1PR1-AC 5′-ACTAGCATCCCGGAGGTTAAAG-3′ and 5′-GCTGTTGCTCCCGTTGTGTA-3′; S1PR1-AD 5′-CTACACAACGGGAGCAACAGC-3′ and 5′-ATGTCACAGGTCTTCGCCTT-3′. The composition and frequency of mutations was analyzed using ICE software (Synthego).

### Activation and photo-conversion of OTII-D2 T cells

Approximately 10^7^ cells from pooled LN and spleens from H2B-Dendra2 × Vav-iCre × OTII mice were i.v. transferred to C57BL/6 recipients that also received 10^6^ ova-loaded LPS-matured BMDCs on the following day by s.c. injection into the right footpad. After 4 days, mice were anesthetized with an intraperitoneal injection of ketamine (50 mg/kg body weight) and xylazine (10 mg/kg body weight). The right hind leg was shaved to enable illumination of the skin above the pop LN. The skin was exposed for 90 s to low intensity, non-collimated light from a BlueWave 75 light system (Dymax, Torrington, CT) equipped with a 390/40 bandpass filter as previously described for illumination of Peyer’s patches and LNs^19^. The distance between the light source and the skin was approximately 1 cm and the circular illumination area had a diameter of approximately 1 cm. The efficiency of the photo-conversion was on average 95%.

### Cell labeling and i.l. injection

In some experiments T cells were labeled with Cell Proliferation Dyes eFluor670 or eFluor450 prior i.l. injection. In experiments cells of different genetic origin or cells treated with antibodies or blocking fusion proteins were injected at a 1:1 ratio, the transferred cell populations were alternately stained with the two dyes to exclude effects of the dyes on cell behavior. Injection procedure has been described previously^10^. Briefly, defined cell numbers diluted in phosphate-buffered saline (PBS) were injected with a pico-injector (Harvard apparatus PLI-100) into the afferent lymph vessel draining towards the popliteal LN of anesthetized (ketamine, xylazine) or sacrificed mice (Figs. 3f–h and 8d–f). Injected cells were analyzed by 2PM or immunohistology (see below) while live injection was documented with a Leica MZ16 epifluorescence microscope and LAS V4.5 software. In some experiments, recipients received 10^6^ PFU of the poxvirus MVA subcutaneously in the hind footpad in ketamine/xylazine-anesthesia 3 days prior to the i.l. injection.

### CD4 T cell culture treatment

Lymphocytes were isolated from pooled peripheral LNs and spleen of donor mice. CD4^+^ T cells were enriched to a purity of 85–95% by AutoMACS with a MACS CD4 negative isolation Kit II (Miltenyi Biotec) and cultured in RPMI 1640 (Gibco) supplemented with 10% heat-inactivated fetal bovine serum (FBS; GE Healthcare Life Sciences, Logan, UT), 2mM l-glutamine, 1% penicillin–streptomycin (both from Gibco) and 50 μM β-mercaptoethanol (Sigma Aldrich). For in vitro activation CD4^+^ T cells were cultured in 96-well plates coated with 0.5 μg/ml anti-CD3 (clone 17A2, prepared in house) and 1 μg/ml anti-CD28 (clone 37.51, eBioscience) antibodies in medium supplemented with Human IL-2 (100 U/ml; Sigma Aldrich) for 2 days at 37 °C with 5% CO_2_. After 2 days, cells were washed and cultured in medium supplemented with IL-2 on non-coated cell culture dishes overnight. For in vivo activation lymphocytes were isolated from peripheral LNs and spleen of GFP-OTII or DO11.10 or DO11.10CCR7^*−*/*−*^ mice and 1  × 10^6^ CD4^+^ T cells were injected intravenously into the tail vein of B6 recipients. One day later mice were immunized intraperitoneally with 5 mg OVA in complete Freund’s adjuvant. After 72 h, lymphocytes were isolated from spleen and peripheral LNs and GFP-CD4^+^ or CD4^+^ TCR OVA^+^ cells were FACS-sorted.

In some experiments cells were incubated with 100 ng/ml PTX or as a control with PBS in RPMI 1640 supplemented with 1% FCS and 25 mM HEPES (Roth) for 2 h at 37 °C. In other experiments cells were incubated with 2% PFA on ice for 15 min. After treatment cells were washed twice before used in subsequent procedures.

For L-Selectin blocking experiments, cells were treated with anti-L-Selectin (MEL-14, 10 µg) or isotype control antibody (1H4, 10 µg) 30 min at 4 °C before i.l. transfer. For PLVAP blocking experiments, anti-PLVAP (MECA-32, 15 µg) or isotype control antibody (CAD9 6H8, 15 µg) were injected s.c. into the hind footpad of anesthetized recipients. For P-Selectin, E-Selectin, and mannan blocking experiments, cells were treated with 12.5 µg fusion protein or 100 µg mannan 30 min at 37 °C before i.l. injection.

### Viral transduction and sorting of targeted T cells

For CD4^+^ T cell transduction, lentiviral particles were first bound to a 96-well plate (Sarstedt) pre-coated with 50 μg/ml Retronectin (Takara, Clontech) by centrifugation (2 h, 2000 × *g*, 4 °C). After removing the supernatant, CD4^+^ T cells (2 ×10^5^) pre-activated for 48 h at 1.3 × 10^6^ cells/ml supplemented with 100 U/ml IL-2 were added, centrifuged (1 h, 700 × *g*, 32 °C), and incubated at 37 °C with 5% CO_2_. After 1 day, cells were collected and fed daily with an equal amount of fresh medium supplemented with 100 U/ml IL-2 and incubated at 37 °C with 5% CO_2_ for 5 days. Cell sorting was performed on a FACSAria Fusion (BD Biosciences) equipped with a ceramic nozzle of 100 μm size and analyzed with FACSDiva (BD Biosciences) and FlowJo software (TreeStar). Pre-gating was used for selecting viable cells and excluding doublets. Cells transduced with lentiviral particles expressing dTomato or eYFP were harvested and immediately sorted. Cells co-transduced with viral particles expressing Cerulean and eYFP were additionally stained with anti-β1 (CD29), β2 (CD18), integrin β7, and αv (CD51) antibodies before cell sorting.

### Immunohistology

LNs (fixed with 2% PFA plus 30% (vol/vol) sucrose and washed with PBS) were embedded in OCT (Tissue-Tek, Sakura) and frozen on dry ice. Eight-micrometer-thick sections were rehydrated and incubated with appropriate blocking reagents and antibodies. In some experiments the GFP or YFP signal of adoptively transferred cells was amplified using a GFP booster (Chromotec). High-resolution composite images were acquired with a Zeiss Axiovert 200M microscope and processed with AxioVision 4.6 software. All pictures were contrast adjusted.

### Quantification of cell migration distance to SCS

Quantification of the cell position in relation to the SCS has been described previously^24^. Briefly, LN boundaries were outlined manually for each image while transferred cells were semi-automatically tracked using the spot detection function of Imaris. Next, ImageJ plugins “analyze particles” and “line graph” were used to determine coordinates of the SCS and transferred cells. The shortest distance of each transferred cell to the SCS was determined by programming a “macro” in Visual Basic for Applications in Microsoft Excel. Several slides of each LN were analyzed. The ratio of cell number was corrected to the exact composition of the input mixture measured by flow cytometry. Based on their positioning within the LN adoptively transferred cells were allocated to three groups (SCS, medullary sinus, LN parenchyma). In some experiments, cells residing in SCS and medullary sinus were pooled and are shown as “cells in sinus system”. SCS and medullary sinus were determined by location and staining with anti-Lyve1 antibodies. The shortest distance of transferred cells to the SCS was only determined for cells residing in the parenchyma.

### Two-photon microscopy

For in vivo imaging, the popliteal LN was surgically exposed and imaged^68,69^. Briefly, for in vivo imaging mice were anesthetized with ketamine and medetomidine and the right leg was fixed in a stretched position. Next, the popliteal LN was gently exposed without compressing the afferent lymphatic vessels. During the entire imaging session, the LN was covered with PBS under a glass coverslip and the local temperature was measured and kept at 37 °C. For two-photon imaging of explanted LNs, LNs were placed in a custom-built incubation chamber at 37 °C. The LNs were superfused with oxygenated (95% O_2_, 5% CO_2_) RPMI medium, as described. In some experiments, Prox1-mOrange2 mice were used as recipients. Structures within the SCS were revealed by s.c. injection of 30 µl 150 µM TAMRA before imaging.

Imaging was performed using an Olympus BX51 upright microscope equipped with a ×20/0.95NA water immersion objective (TriM Scope, LaVision Biotec, Bielefeld, Germany). For two-photon excitation, Mai Tai HP tunable lasers were used (Spectra Physics). To excite eGFP, RFP, or TAMRA, the Mai Tai laser was tuned to 920 or 865 nm, respectively. For visualization of mOrange2 and eFluor 660, an optical parametric oscillator (APE, Berlin) was used with the output beam tuned to 1100 nm. Alternatively, an InSight DS laser (Spectra Physics) was used. Two-photon imaging data was analyzed using Imaris 7.x-8.x (Bitplane). All movies and z-stacks were median-filtered. 3D drift correction was applied, when needed. Tracking was done automatically with manual corrections.

### Confocal microscopy

Mice were anesthetized with ketamine and xylazine before s.c. injection of 30 µl of anti-mouse laminin antibody. After 10 min, labeled cells were adoptively i.l. transferred and the LNs were excised and fixed within 30 min. Fifty-micrometer-thick sections were counterstained with antibodies in a wet chamber immediately after sectioning. Images were acquired using Olympus FluoView 1000 and Leica inv-3 and contrast adjusted with Imaris 7.x-8.x (Bitplane).

### Statistical analysis

Statistical analysis was performed with Prism 4 (Graph-Pad Software, Inc.). Statistical significance for paired data sets were calculated using the two-tailed non-parametric Wilcoxon signed rank test and for unpaired data sets the non-parametric Mann Whitney test. Effect sizes depicted in Figs. 6–8 were calculated based on medians.

### Reporting summary

Further information on research design is available in the Nature Research Reporting Summary linked to this article.

## Supplementary information

Supplementary Information

Reporting Summary

Description of Additional Supplementary Information

Supplementary Movie 1

Supplementary Movie 2

Supplementary Movie 3

Supplementary Movie 4

Supplementary Movie 5

Supplementary Movie 6

Supplementary Movie 7

## Data Availability

The source data underlying Fig. 1b, c, 3b, c, e, 4b, c, 5a–d, 6a–d, 7a–c, 8a–f, and Supplementary Figs. 2c, 5a–c, 6a–b, and 8a–c, are provided as a Source Data file. All other data that support the findings of this study are available from the corresponding author upon reasonable request.

## Source data

Source data
