# Supplementary material for: Efficient homing of T cells via afferent lymphatics requires mechanical arrest and integrin-supported chemokine guidance
Source: Nat Commun. 2020 Feb 28;11:1114. doi: 10.1038/s41467-020-14921-w (PMC7048855; doi:10.1038/s41467-020-14921-w)
Supplement: Supplementary file 1 — Supplementary Information [file 41467_2020_14921_MOESM1_ESM.pdf]

SUPPPLEMENTARY INFORMATION

Martens et al., Efficient homing of activated T cells via afferent lymphatics requires mechanical arrest and integrin-supported chemokine guidance

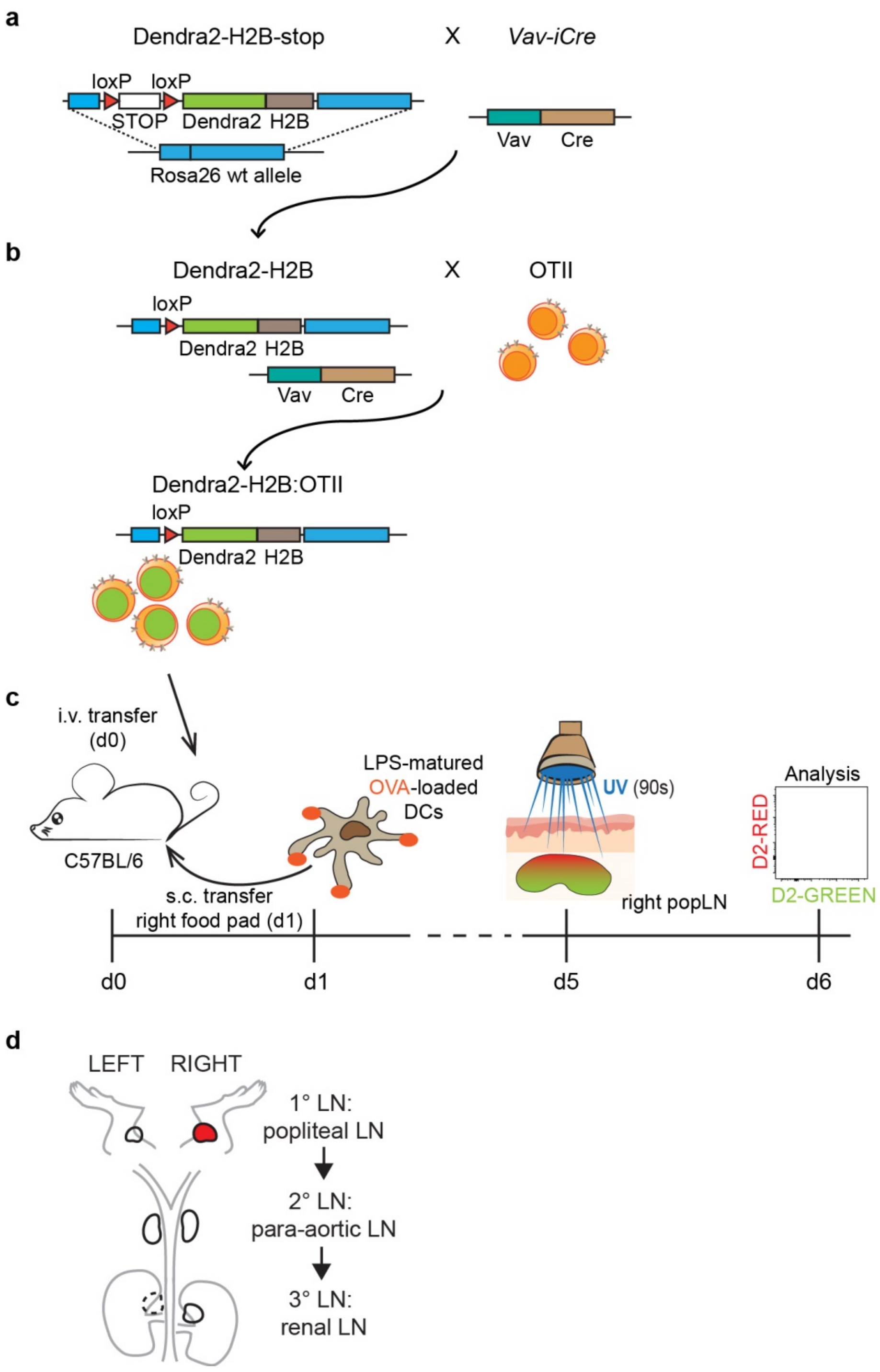

Supplementary Figure 1. Schematic representation of the experimental work flow. (a) A fusion protein of the photoconvertible dye Dendra2 and histone H2B with an upstream flox-stop-flox-cassette is transgenically expressed in the Rosa-26 locus of the new mouse strain Dendra2-H2B-stop. Intercrossing of these mice with Vav-Cre leads to the deletion of the stop-cassette in cells of hematopoietic and endothelial origin. **(b)** Offspring of the Dendra2-H2B-stop x Vav-Cre breeding was crossed to OTII mice in order to generate mice expressing photoconvertible

Dendra2-GREEN fluorescent protein in antigen-specific CD4 T cells (OTII-Dendra2). **(c)** OTII-Dendra2 cells were injected i.v. into C57BL/6 recipients one day prior to s.c. immunization with LPS-matured OVA-loaded DCs into the right foot pad. At day 5, fur from the right popliteal fossa was removed and the skin area above the right pop LN was exposed to UV-light for 90 sec. converting the green fluorescent protein Dendra2 (D2-GREEN) into a red fluorescent protein (D2-RED). LNs were analyzed for D2-GREEN and D2-RED fluorescence on day 6. **(d)** Schematic drawing of lymph flow from the 1° popliteal LNs to downstream 2° para-aortic and 3° renal LNs. The photo-converted right popLN is depicted in red. Please note that the left renal lymph node is not present in every mouse.

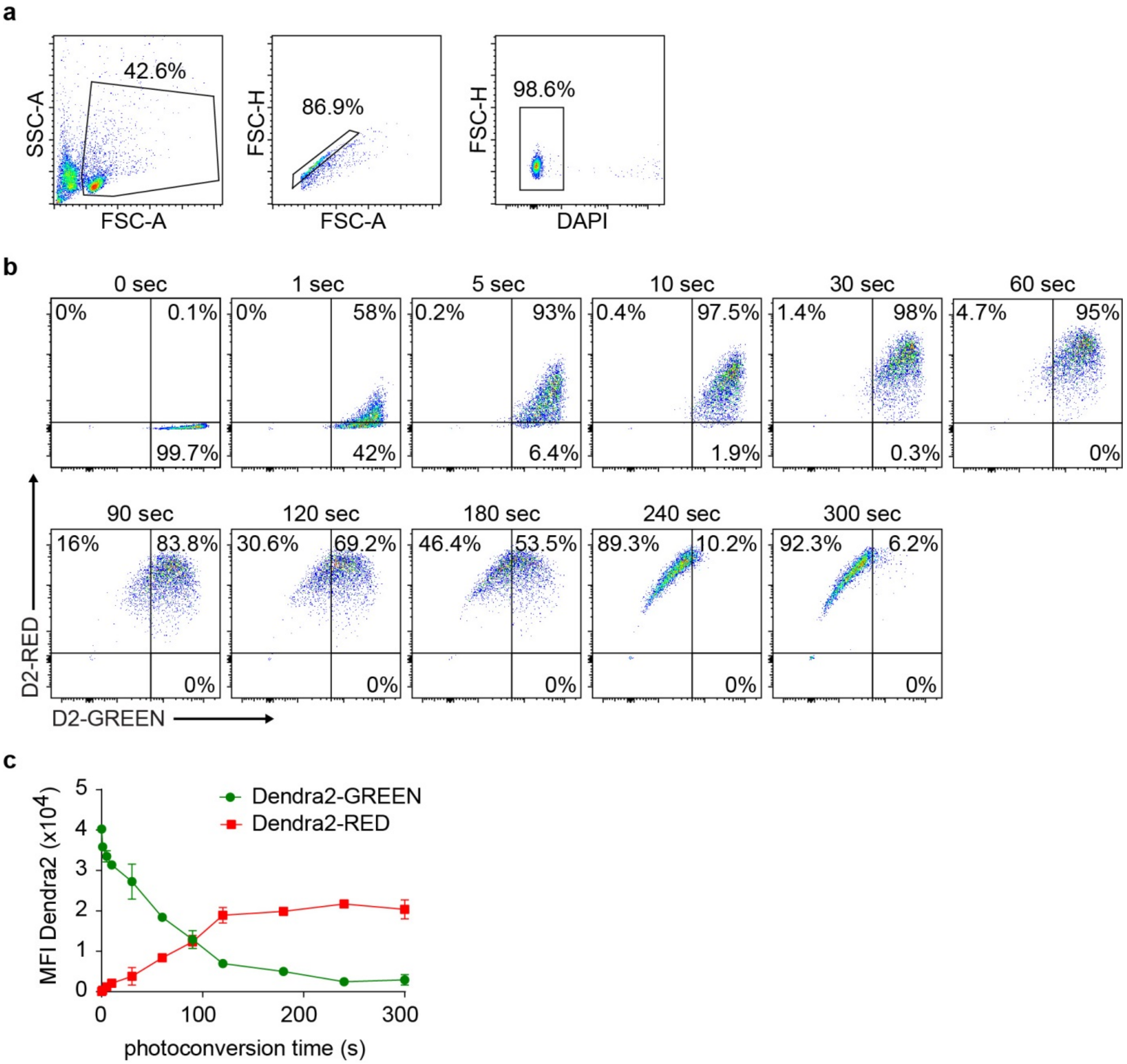

Supplementary Figure 2. Dendra2 photoconversion *in vitro*. 6-10 x 10^3 cells in suspension, isolated from pooled lymph nodes of a Dendra2-H2B-Vav-iCre mouse, were photo-converted for different times with an UV light source. Photo-conversion was measured by flow cytometry. **(a)** Gating strategy of live lymphocytes. **(b)** Time kinetics of Dendra2 photo-conversion from Dendra2-green to Dendra2-red of live lymphocytes. **(c)** Quantification of mean fluorescence intensity (MFI) of Dendra2-green and Dendra2-red signals after different times of photo-conversion. Data are representative of two independent experiments.

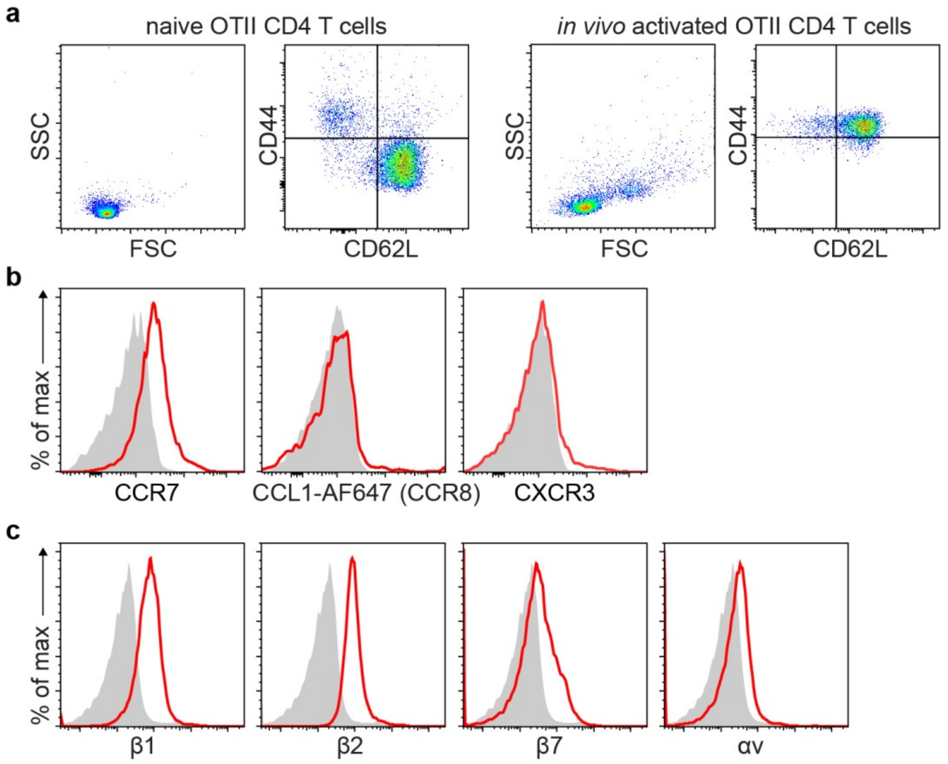

Supplementary Figure 3. ***In vivo*** **activated CD4 T cells express CCR7 and homing receptors. (a)** Representative FACS plots showing the forward and side scatter of naïve (left) and *in vivo* activated (right) OTII $CD4^+$ T cells as well as the expression of CD62L and CD44. **(b)** Expression of chemokine receptors on *in vivo* activated OTII $CD4^+$ T cells (red lines); grey shaded areas, isotype control (CCR7 and CXCR3) or FMO (CCR8). **(c)** Expression of integrin subunits β1 (CD29), β2 (CD18), β7, and αv (CD51) on *in vivo* activated OTII $CD4^+$ T cells (red lines); grey shaded areas, isotype control.

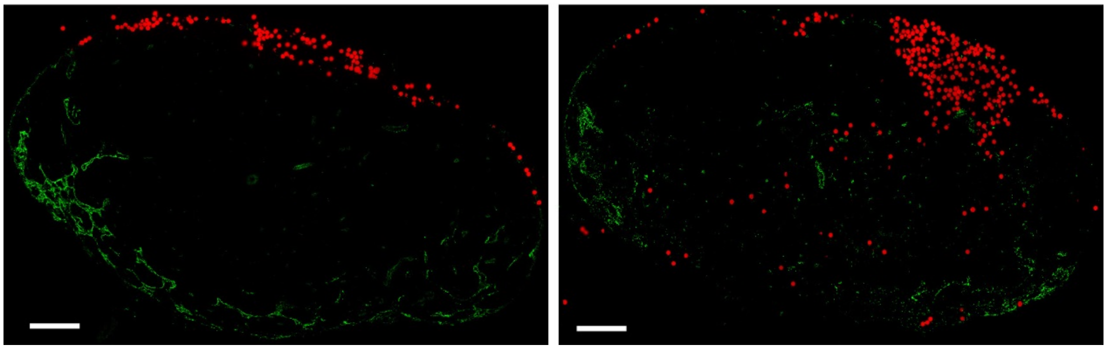

Supplementary Figure 4. Injection of 15 µm beads leads to the disruption of the SCS floor in inflamed LNs. In order to facilitate i.l. injection of 15 µm latex beads, the injection pressure had to be increased from 15 - 25 kPa (used in standard injections) to 100 kPa, which lead to an aberrant accumulation of particles within the SCS and interfollicular areas. Immunohistology of inflamed pop LNs directly after i.l. injection of fluorescent latex particles. Two representative images of 6 LNs from two independent experiments are shown. Scale bar 100 µm.

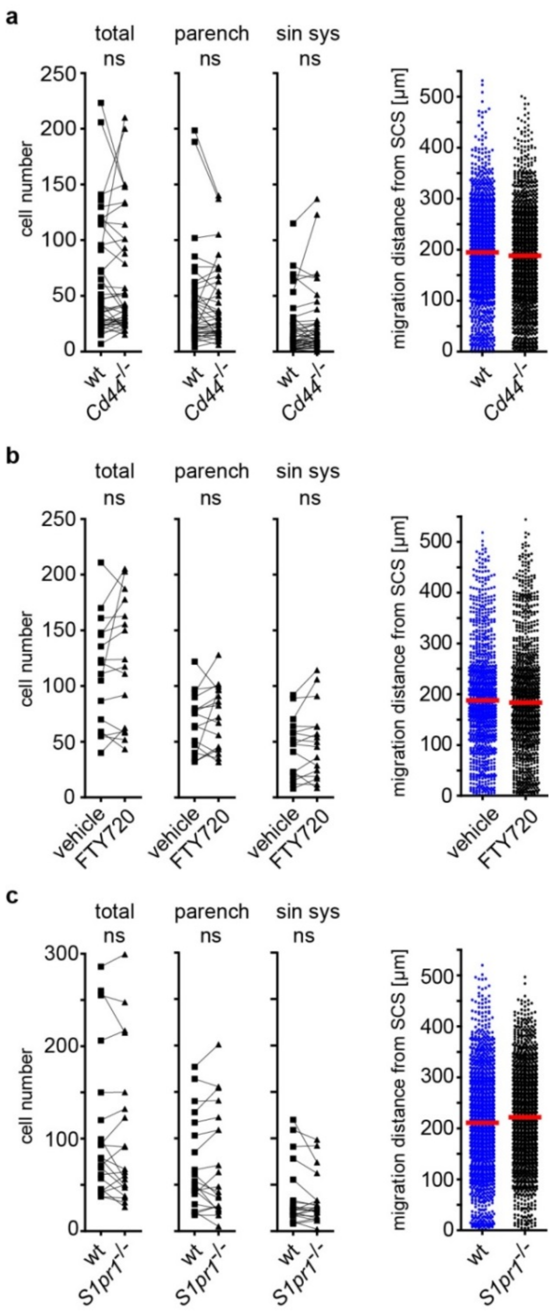

Supplementary Figure 5. CD44 and S1PR1 do not contribute to homing of lymph-derived activated CD4 T cells to LNs. Quantitative analysis of adoptively transferred cells in popliteal LNs 90 min after i.l. injection of combinations of activated $CD4^+$ T cells as indicated; **(a)** wild-type and *Cd44*$^{-/-}$; **(b)** wild-type and FTY720-treated cells; **(c)** wild-type and *S1pr1*$^{-/-}$. Left, total cell counts; dots represent cell counts per LN section; (parench, parenchyma; sin. sys., sinus system). Right, migration distance from the SCS; dots represent the shortest distance of each cell to the SCS; red bars, median; ns, not significant; Wilcoxon signed rank test. Data derived from 4 – 8 LNs with 4 mice analyzed per group.

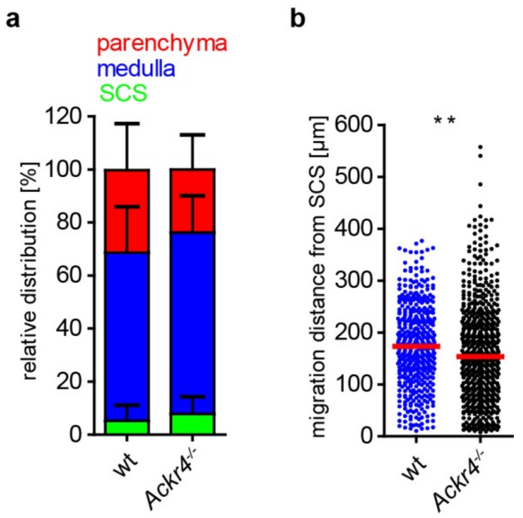

Supplementary Figure 6. ACKR4 expression on endothelial cells of the SCS affects homing of lymph-derived activated CD4 T cells. Relative distribution **(a)**, and migration distance from the SCS **(b)** of *in vivo* activated OT II T cells 90 min after i.l. injection into popliteal LNs of WT or *Ackr4* deficient recipients as indicated; **(b)** dots represent the shortest distance of each cell to the SCS; Data are representative of three independent experiments with a total of 8 – 11 LNs analyzed. Error bars, SD; red bars, median; Mann Whitney test; **, $p < 0.01$.

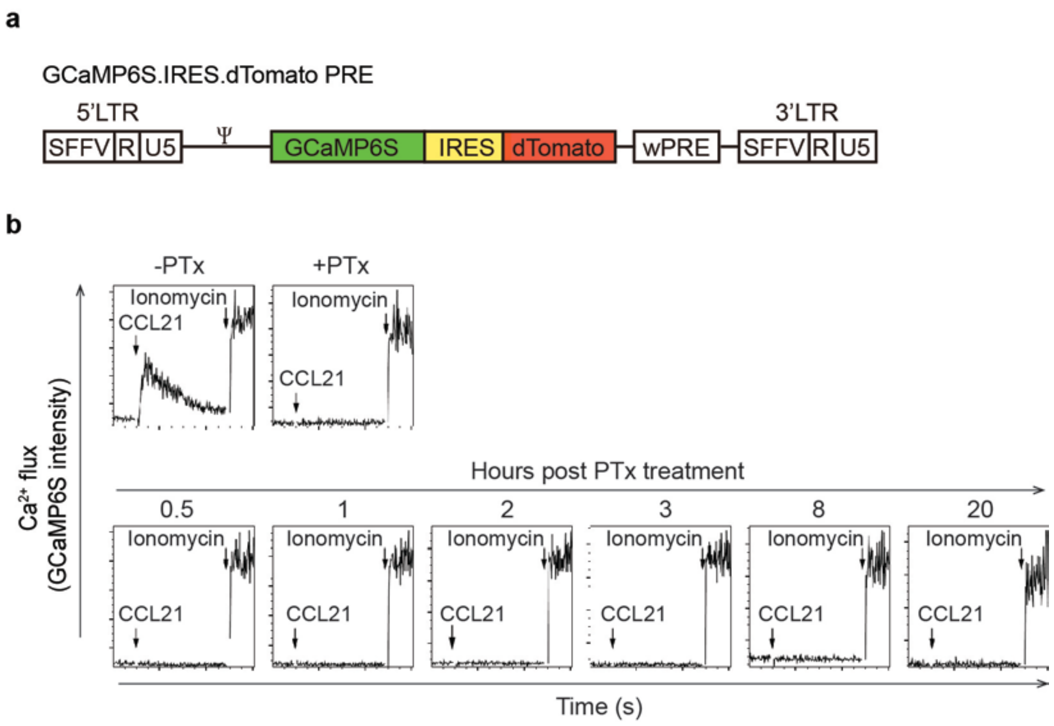

Supplementary Figure 7. Pertussis toxin treatment inhibits CCL21-induced Ca^{2+} influx in activated CD4 T cells for more than 20 hr. (a) Retroviral vector used to transduce cells with GCaMP6s and the reporter gene dTomato. **(b)** Activated $CD4^+$ T cells transduced with a retrovirus expressing the calcium sensor GCaMP6s were treated with 100 ng/ml of PTX at 37°C for 2hr or left untreated (- PTX). After washing GCaMP6s intensity was measured immediately (Upper) or at indicated time-points after PTX treatment (Lower) by flow cytometry. After 45s to establish a baseline, cells were treated with 500 ng/ml CCL21 and signals were recorded for additional 3 minutes 15 seconds, when 1 µg/ml of ionomycin was added and signals were recorded for additional 1 min. Data are the representative of 2 independent experiments.

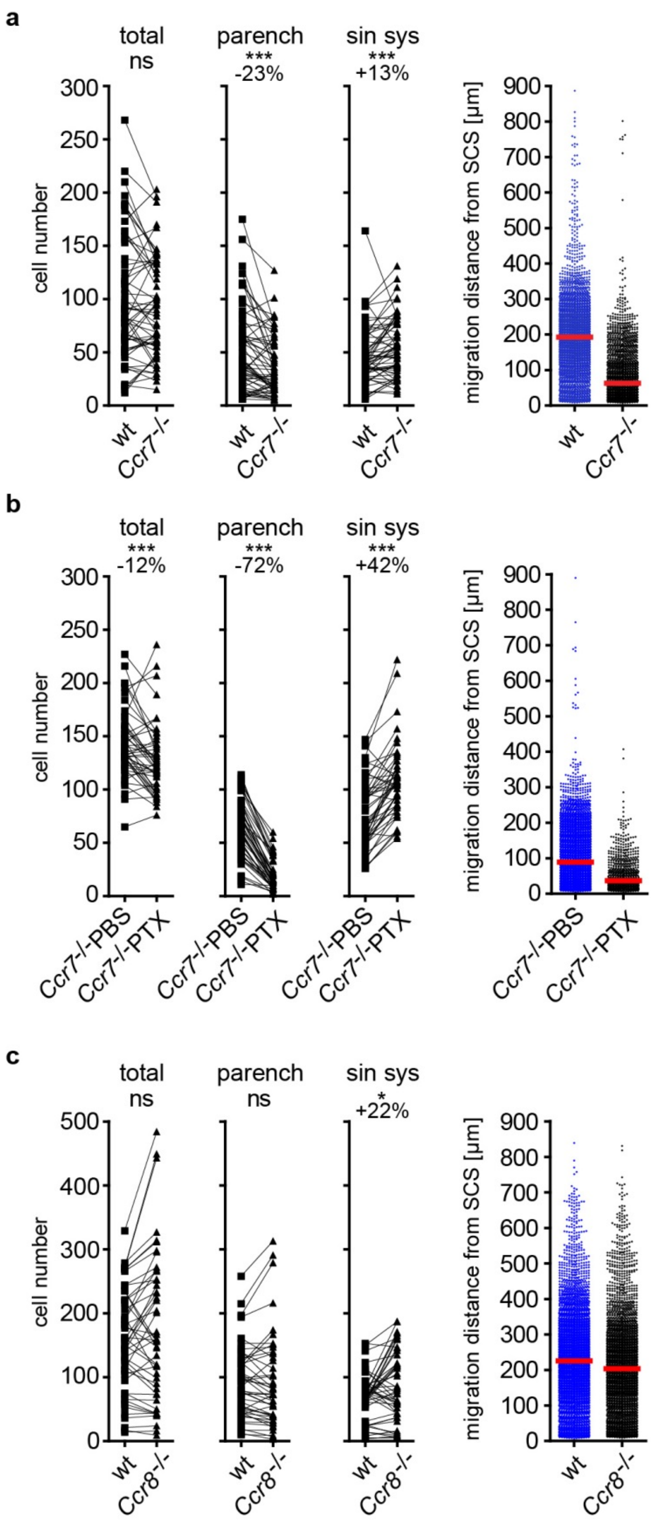

Supplementary Figure 8. Chemokine dependent entry of lymph-derived activated CD4 T cells into inflamed LNs. 3 days after subcutaneous MVA infection, activated wild-type and *Ccr7*$^{-/-}$ **(a)**; PBS-treated *Ccr7*$^{-/-}$ and PTX-treated *Ccr7*$^{-/-}$ **(b)** and wild-type and *Ccr8*$^{-/-}$ **(c)** CD4^{+} T cells (8 x 10^{5} total cells; 1:1 mixture) were adoptively transferred by i.l. injection into popliteal LNs. Left, total

cell counts; dots represent cell number per LN section; numbers above indicate percentage of change compared to control population; (parench, parenchyma; sin. sys., sinus system); ns, not significant; Wilcoxon signed rank test *, $p < 0.05$; ***, $p < 0.001$. Right, migration distance from the SCS; dots represent the shortest distance of each cell to the SCS; red bars, median. Data are representative of 3 independent experiments with a total of 15 LNs (a) or 2 independent experiments with a total of 12 LNs (b, c).

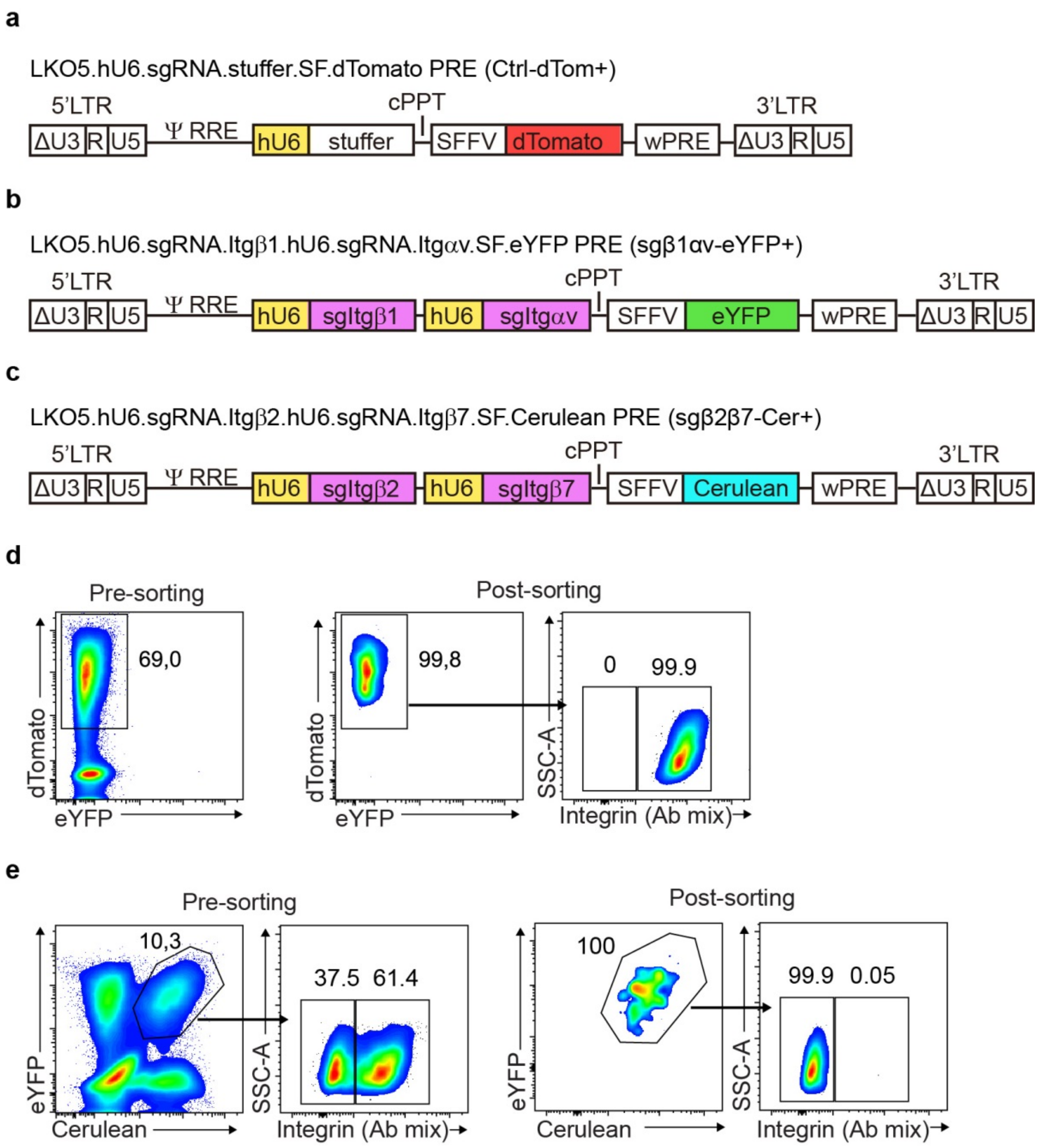

Supplementary Figure 9. Generation of CRISPR/Cas9-mediated integrin knockout CD4 T cells with sgRNA-expressing lentiviral vectors. (a-c) Vectors are based on the pLKO.5 lentiviral backbone and contain two LTRs with deleted U3 regions (ΔU3, self-inactivating design). Expression of single guide RNAs (sgRNA) is driven by the human U6 Pol III promoter (hU6) and the expression of the fluorescent reporter from spleen focus-forming virus promoter (SFFV). Vectors lacking the complementary 20nt targeting sequence but containing the crRNA and tracrRNA elements were used as a control. Vectors simultaneously expressing two sgRNA were generated by inserting a second hU6 promoter / sgRNA expression cassette behind the first one. **(a)** Vector expressing dTomato as a fluorescent reporter was used as a control. **(b)** β1 and av sgRNA were cloned into a vector expressing eYFP as a fluorescent reporter. **(c)** β2 and β7 sgRNA were cloned into a vector expressing Cerulean as a fluorescent reporter. Ψ: retroviral packaging signal; RRE: Rev responsive element; cPPT: central polypurine tract; wPRE: post transcriptional

element from woodchuck hepatitis virus; R: redundant region; U5: unique 5 region. **(d,e)** FACS analysis of activated $CD4^+$ T cells 5 days after transduction with Ctrl-$dTom^+$ **(d)** or after co-transduction with sgβ1αv-$eYFP^+$ together with sgβ2β7-Cer^+ **(e)** virus before (left) and after (right) cell sorting. Upon co-transduction, $CD4^+$ T cells simultaneously targeted against *Itgb1*, *Itgb2*, *Itgb7*, *Itgav* were obtained by sorting double positive cells ($eYFP^+Cerulean^+$) followed by negative selection of integrin expressing cells after staining with β1, β2, β7, av integrin antibodies at the same time (antibody mix). Simultaneous expression of all 4 integrins was assessed in sorted cells with the same antibody mix (right). The gating strategy shown in (d) and (e) was used to sort control or $4Itg^{-/-}$ cells, respectively, presented on Fig. 8a,c,e,f.

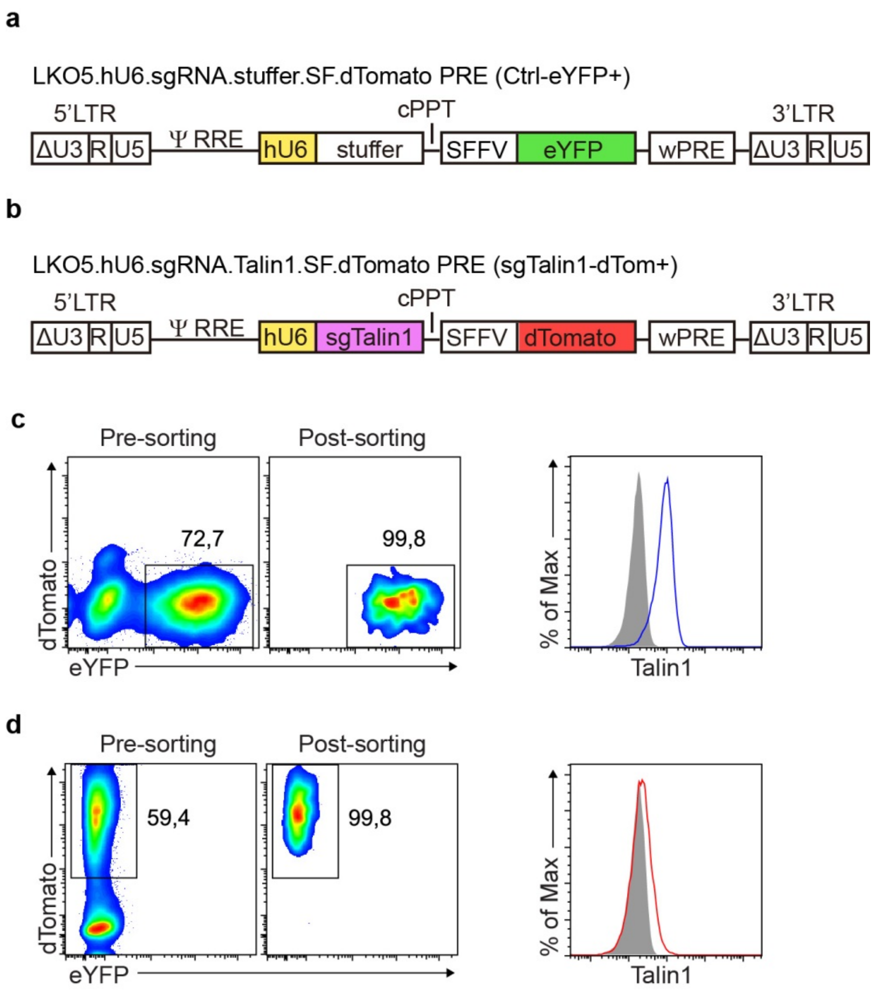

Supplementary Figure 10. Generation of CRISPR/Cas9-mediated *Talin1* knockout CD4 T cells with sgRNA-expressing lentiviral vectors. (a) Vector expressing eYFP as a fluorescent reporter and without a functional sgRNA was used as a control. **(b)** Talin1 sgRNA was cloned into a vector expressing dTomato as a fluorescent reporter. **(c,d)** FACS analysis of activated $CD4^+$ T cells 5 days after transduction with Ctrl-$eYFP^+$ **(c)** or with sgTalin1-$dTom^+$ **(d)** virus before (left) and after (middle) cell sorting. Expression of Talin1 was assessed in sorted cells (right); grey shaded curves, isotype control. The gating strategy shown in (c) and (d) was used to sort control or *Talin1*$^{-/-}$ cells, respectively that were used for experiments shown in Fig. 8b-d.

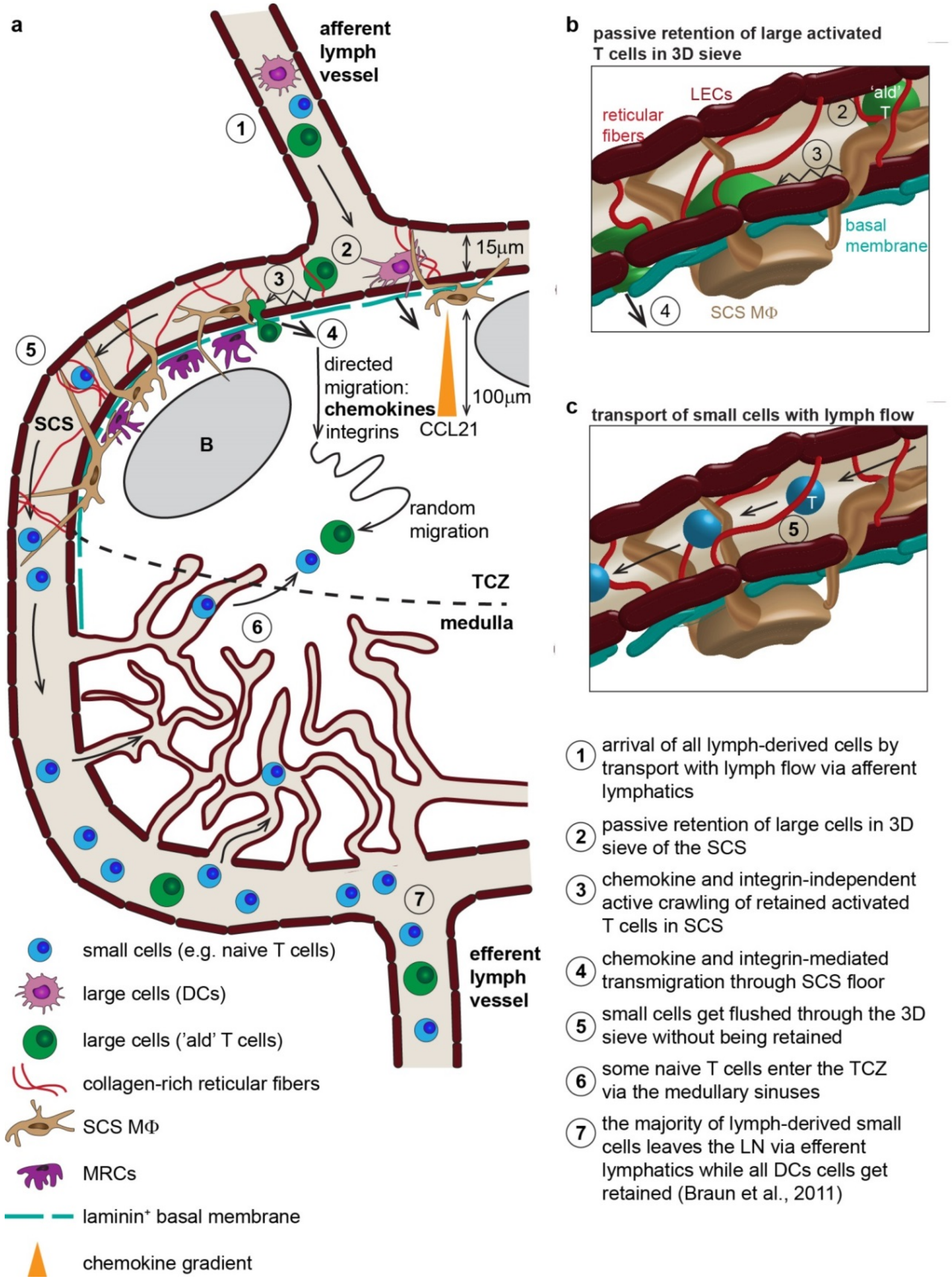

Supplementary Figure 11. The SCS 3D sieve retains large cells while small cells are transported through by lymph flow. (a) 1. Lymph-derived cells arrive in the LN with the lymph flow via afferent lymphatics. 2. Large cells like 'ald' T cells or DCs get passively retained in the 3D sieve of

the SCS. 3. Retained activated T cells start crawling on the SCS ECs in a chemokine- and integrin-independent manner. 4. They transmigrate through the SCS ECs into the LN parenchyma which is chemokine- and to a certain degree also integrin-driven. Subsequently, transmigrated T cells migrate along an approx. 100 µm long chemokine gradient towards the TCZ where they then start to migrate randomly. 5. Smaller cells like naïve T cells are transported with lymph flow towards the medulla as they can pass through the pores of the 3D sieve. 6. Some naïve cells enter the TCZ via the medullary sinus system. 7. The majority of naïve T cells and a few activated T cells leave the LN via the efferent lymphatics while all DCs get retained in the SCS (Braun et al., 2011). **(b)** Reticular fibers and cellular processes or cell bodies from presumably SCS MΦ create a 3D environment within the SCS. 2. Large activated T cells are passively retained within these structures. 3. They subsequently start crawling on the ECs of the SCS floor and subsequently transmigrate into the LN parenchyma. The laminin$^+$ basal membrane contains pores which serve as entry ports for transmigrating T cells. **(c)** Smaller cells are transported with the lymph flow. They fit through the 3D environment of the SCS without being retained.
‘ald’: activated lymph-derived; B: B cell follicle; DCs: dendritic cells; ECs: endothelial cells; MRCs: marginal reticular cells; MΦ: macrophage; SCS: subcapsular sinus; TCZ: T cell zone
