# Supplementary material for: Efficient homing of T cells via afferent lymphatics requires mechanical arrest and integrin-supported chemokine guidance
Source: Nat Commun. 2020 Feb 28;11:1114. doi: 10.1038/s41467-020-14921-w (PMC7048855; doi:10.1038/s41467-020-14921-w)
Supplement: Supplementary file 2 — Reporting Summary [file 41467_2020_14921_MOESM2_ESM.pdf]

nature research

Corresponding author(s): Reinhold Förster

Last updated by author(s): Jan 29, 2020

Reporting Summary

Nature Research wishes to improve the reproducibility of the work that we publish. This form provides structure for consistency and transparency in reporting. For further information on Nature Research policies, see Authors & Referees and the Editorial Policy Checklist .

Statistics

For all statistical analyses, confirm that the following items are present in the figure legend, table legend, main text, or Methods section.

n/a	Confirmed	
☐	☒	The exact sample size (*n*) for each experimental group/condition, given as a discrete number and unit of measurement
☐	☒	A statement on whether measurements were taken from distinct samples or whether the same sample was measured repeatedly
☐	☒	The statistical test(s) used AND whether they are one- or two-sided<br>*Only common tests should be described solely by name; describe more complex techniques in the Methods section.*
☐	☒	A description of all covariates tested
☐	☒	A description of any assumptions or corrections, such as tests of normality and adjustment for multiple comparisons
☐	☒	A full description of the statistical parameters including central tendency (e.g. means) or other basic estimates (e.g. regression coefficient) AND variation (e.g. standard deviation) or associated estimates of uncertainty (e.g. confidence intervals)
☐	☒	For null hypothesis testing, the test statistic (e.g. *F*, *t*, *r*) with confidence intervals, effect sizes, degrees of freedom and *P* value noted<br>*Give P values as exact values whenever suitable.*
☒	☐	For Bayesian analysis, information on the choice of priors and Markov chain Monte Carlo settings
☒	☐	For hierarchical and complex designs, identification of the appropriate level for tests and full reporting of outcomes
☒	☐	Estimates of effect sizes (e.g. Cohen's *d*, Pearson's *r*), indicating how they were calculated

Our web collection on statistics for biologists contains articles on many of the points above.

Software and code

Policy information about availability of computer code

Data collection	*Provide a description of all commercial, open source and custom code used to collect the data in this study, specifying the version used OR state that no software was used.*
Data analysis	*Provide a description of all commercial, open source and custom code used to analyse the data in this study, specifying the version used OR state that no software was used.*

For manuscripts utilizing custom algorithms or software that are central to the research but not yet described in published literature, software must be made available to editors/reviewers. We strongly encourage code deposition in a community repository (e.g. GitHub). See the Nature Research guidelines for submitting code & software for further information.

Data

Policy information about availability of data

All manuscripts must include a data availability statement. This statement should provide the following information, where applicable:
- Accession codes, unique identifiers, or web links for publicly available datasets
- A list of figures that have associated raw data
- A description of any restrictions on data availability

The source data underlying Figs. 1b, c, 3b, c, e, 4b, c, 5a-d, 6a-d, 7a-c, 8a-f, and Supplementary Figs. 2c, 5a-c, 6a-b, and 8a-c, are provided as a Source Data file. All other data that support the findings of this study are available from the corresponding author upon reasonable request.

Field-specific reporting

Please select the one below that is the best fit for your research. If you are not sure, read the appropriate sections before making your selection.

Life sciences study design

All studies must disclose on these points even when the disclosure is negative.

Sample size	To ensure enough statistical power, we chose the sample size based on estimates from preliminary results as well as on our previous publications performing similar analysis.
Data exclusions	No data were excluded
Replication	All experiments were repeated at least three times. In some cases, when sample size was large enough, two replicate experiments were performed.
Randomization	No randomization was used
Blinding	Quantitative analysis of transfered cells in lymph nodes using histology sections was blinded.

Reporting for specific materials, systems and methods

We require information from authors about some types of materials, experimental systems and methods used in many studies. Here, indicate whether each material, system or method listed is relevant to your study. If you are not sure if a list item applies to your research, read the appropriate section before selecting a response.

Materials & experimental systems

n/a	Involved in the study	
☐	☒	Antibodies
☐	☒	Eukaryotic cell lines
☒	☐	Palaeontology
☐	☒	Animals and other organisms
☒	☐	Human research participants
☒	☐	Clinical data

Methods

n/a	Involved in the study	
☒	☐	ChIP-seq
☐	☒	Flow cytometry
☒	☐	MRI-based neuroimaging

Antibodies

Antibodies used	All antibodies are listed in the methods, with the clone number and dilution used.
Validation	All antibodies were previously tested and used at concentrations that exhibited the expected staining pattern.

Eukaryotic cell lines

Policy information about cell lines

Cell line source(s)	HEK 293T cells were provided by the Institute of Experimental Hematology (IEH), MHH, Hannover.
Authentication	HEK 293T cells were obtained as per-authenticated by the IEH.
Mycoplasma contamination	HEK 293T cells were negative for Mycoplasma contamination.
Commonly misidentified lines (See ICLAC register)	*Name any commonly misidentified cell lines used in the study and provide a rationale for their use.*

Animals and other organisms

Policy information about studies involving animals; ARRIVE guidelines recommended for reporting animal research

Laboratory animals	C57BL/6, BALB/c, CByJ.B6-Tg(CAG-EGFP)1Osb/J (Stock No: 007075), B6Cg-Tg(TcraTcrb)425Cbn/J (Stock No: 004194),B6.Cg-Commd10Tg(Vav1-icre)A2Kio/J (Stock No: 008610), B6.129P2(C)-Ccr7tm1Rfor/J, B6.129P2/Sv-Ccr5tm1Kuz/J (Stock No: 005427), BALB/c-Tg(DO11.10)10Loh/J (Stock No: 003303), B6.129P2-Cxcr3tm1/Raks, B6.129P-CCR8tm1/Lira, B6.129 prox1-mOrange2-pA-BAC, B6.129S4-Icam1tm1Jcgr/J (Stock No: 002867), B6J.129(Cg)-Gt(ROSA)26Sortm1.1(CAG-cas9*,-EGFP)Fezh/J (Stock No: 026179), B6.129S6 Ackr4tm1.1Rjbntm1. Gt(ROSA)26Sortm1(LSL-H2B-Dendra2) was generated by Cyagen.
Wild animals	This study did not involve wild animals.
Field-collected samples	This study did not involve samples collected from the field.

Ethics oversight

All experiments were conducted in accordance with the local animal welfare regulations reviewed by the institutional review board and the Niedersächsisches Landesamt für Verbraucherschutz und Lebensmittelsicherheit (LAVES).

Note that full information on the approval of the study protocol must also be provided in the manuscript.

Flow Cytometry

Plots

Confirm that:

- [x] The axis labels state the marker and fluorochrome used (e.g. CD4-FITC).
- [x] The axis scales are clearly visible. Include numbers along axes only for bottom left plot of group (a 'group' is an analysis of identical markers).
- [x] All plots are contour plots with outliers or pseudocolor plots.
- [x] A numerical value for number of cells or percentage (with statistics) is provided.

Methodology

Sample preparation	Lymphocytes were isolated from pooled peripheral LNs and spleen of donor mice. CD4+ T cells were enriched to a purity of 85–95% by AutoMACS with a MACS CD4 negative isolation Kit II (Miltenyi Biotec) and cultured in RPMI 1640 (Gibco) supplemented with 10% heat-inactivated fetal bovine serum (FBS; GE Healthcare Life Sciences, Logan, UT), 2mM L-Glutamine, 1% Penicillin-Streptomycin (both from Gibco) and 50 μM β-Mercaptoethanol (Sigma-Aldrich). For CD4+ T cell transduction or nucleofection we used CD4+ T cells (2 x 105) pre-activated for 48h with 0.5 μg/ml anti-CD3 (clone 17A2, prepared in house) and 1 μg/ml anti-CD28 (clone 37.51, eBioscience) antibodies.
Instrument	BD LSRII, BD FACSAria Fusion
Software	BD FACSDiva and FlowJo software
Cell population abundance	For FACS sorting, purity was usually >95% and was determined by applying the same gating strategy used during sorting. For FACS, typically 10.000-20.000 events were recorded from the population of interest.
Gating strategy	Pre-gating was used for selecting viable cells and excluding doublets. CD4+ T cells simultaneously targeted against Itgb1, Itgb2, Itgb7, Itgav were obtained by sorting double positive cells (eYFP+Cerulean+) followed by negative selection of integrin expressing cells after staining with β1, β2, β7, av integrin antibodies at the same time. Simultaneous expression of all 4 integrins was assessed in sorted cells with the same antibody mix applying the same gating strategy. Cells transduced with lentiviral particles expressing dTomato (Talin1 knockout) or eYFP (control cells) were harvested and immediately sorted 5 days after transduction.

- [x] Tick this box to confirm that a figure exemplifying the gating strategy is provided in the Supplementary Information.
