# Supplementary material for: Efficient homing of T cells via afferent lymphatics requires mechanical arrest and integrin-supported chemokine guidance
Source: Nat Commun. 2020 Feb 28;11:1114. doi: 10.1038/s41467-020-14921-w (PMC7048855; doi:10.1038/s41467-020-14921-w)
Supplement: Supplementary file 3 — Description of Additional Supplementary Information [file 41467_2020_14921_MOESM3_ESM.pdf]

Description of Additional Supplementary Files

File Name: Supplementary Movie 1
Description: Initial retention of cells in the SCS is independent of adhesion molecules. Epifluorescence live imaging of the popliteal LN while i.l. injection of 4-5 x 104 untreated or PFA-fixed 2 in vitro activated CD4 T cells or 6 µm latex beads. Cells and particles float freely in before they are instantaneously arrested within the SCS.

File Name: Supplementary Movie 2
Description: Homing of lymph-derived activated T cells. To study homing from the SCS by 2PM activated CD4 T cells (green) were i.l. delivered and imaged ex vivo. LN capsule visualized by SHG (blue). Part 1: Within the SCS cells migrate slowly and randomly (top view, white tracks). Part 2: After crossing the SCS floor cells directionally migrate towards the T cell zone for distances of approximately 100 µm (side view, green tracks) before starting random walk migration (red tracks). Scale bar, 50 µm.

File Name: Supplementary Movie 3
Description: Migration within the SCS but not translocation into the lymph node parenchyma occurs independent of chemokine receptors. Unimpaired crawling of PTX-treated wt in vitro activated CD4 T cells on the SCS floor). However, PTX-treated cells only occasionally enter the LN parenchyma but do not directionally translocate to the TCZ. PTX-treated activated T cells (green); SHG, blue. Scale bar, 20 µm.

File Name: Supplementary Movie 4
Description: In vivo activated OTII cells can home through portals in the SCS floor. In vivo activated OTII cells (green) enter the lymph node parenchyma through the same "entry ramp" (green tracks) after i.l. transfer into a Prox-1-mOrange2 (red) recipient. In vivo 2PM movie. SHG, blue.

File Name: Supplementary Movie 5
Description: Migration of Talin-1-/- T cells. Activated wt (green) or Talin-1-/- (red) T cells show random walk migration within the SCS. SHG, blue.

File Name: Supplementary Movie 6
Description: Migration of activated T cells deficient for four integrin chains. Activated wt T cells (green) or activated T cells deficient for four integrins (Itgb1-/- , Itgb2-/- , Itgb7-/- , and Itgav-/- ; 4Itg-/- , red) show random walk migration within the SCS. SHG, blue.

File Name: Supplementary Movie 7
Description: Migration of activated T cells deficient for four integrin chains and treated with pertussis toxin. Activated wt T cells (green) or activated T cells deficient for four integrins (Itgb1-/- , Itgb2-/- , Itgb7-/- , and Itgav-/- ; 4Itg-/- , red), both treated with pertussis toxin (PTX), show random walk migration within the SCS. SHG, blue.
